# Global HIV mortality trends among children on antiretroviral treatment corrected for under‐reported deaths: an updated analysis of the International epidemiology Databases to Evaluate AIDS collaboration

**DOI:** 10.1002/jia2.25780

**Published:** 2021-09-21

**Authors:** Reshma Kassanjee, Leigh F. Johnson, Elizabeth Zaniewski, Marie Ballif, Benedikt Christ, Constantin T. Yiannoutsos, Patience Nyakato, Sophie Desmonde, Andrew Edmonds, Tavitiya Sudjaritruk, Jorge Pinto, Rachel Vreeman, Désiré Lucien Dahourou, Christelle Twizere, Azar Kariminia, James G. Carlucci, Charles Kasozi, Mary‐Ann Davies, the International epidemiology Databases to Evaluate AIDS (IeDEA) Collaboration

**Affiliations:** ^1^ Centre for Infectious Disease Epidemiology and Research (CIDER) School of Public Health and Family Medicine University of Cape Town Cape Town South Africa; ^2^ Institute of Social and Preventive Medicine (ISPM) University of Bern Bern Switzerland; ^3^ Richard M. Fairbanks School of Public Health Indiana University Indianapolis Indiana USA; ^4^ Centre d'Epidémiologie et de Recherche en santé des Populations (CERPOP) Inserm U1027/University Toulouse 3 Toulouse France; ^5^ Department of Epidemiology Gillings School of Global Public Health The University of North Carolina at Chapel Hill Chapel Hill North Carolina USA; ^6^ Department of Pediatrics Faculty of Medicine Chiang Mai University Chiang Mai Thailand; ^7^ Clinical and Molecular Epidemiology of Emerging and Re‐emerging Infectious Diseases Research Cluster Faculty of Medicine Chiang Mai University Chiang Mai Thailand; ^8^ School of Medicine Federal University of Minas Gerais Belo Horizonte Brazil; ^9^ Department of Global Health and Health System Design Icahn School of Medicine at Mount Sinai New York New York USA; ^10^ Arnhold Institute for Global Health Icahn School of Medicine at Mount Sinai New York New York USA; ^11^ Academic Model Providing Access to Healthcare (AMPATH) Eldoret Kenya; ^12^ Département Biomédical/Santé Publique Institut de Recherche en Sciences de la Santé Ouagadougou Burkina Faso; ^13^ CA‐IeDEA Burundi Centre National de Référence en matière de VIH/SIDA (CNR) Bujumbura Burundi; ^14^ Kirby Institute, UNSW Sydney Sydney Australia; ^15^ Ryan White Center for Pediatric Infectious Diseases and Global Health Indiana University School of Medicine Indianapolis Indiana USA; ^16^ Masaka Regional Referral Hospital Masaka Uganda

**Keywords:** antiretroviral therapy, HIV, mortality, tracing, trends, under‐ascertainment

## Abstract

**Introduction:**

The Joint United Nations Programme on HIV/AIDS (UNAIDS) projections of paediatric HIV prevalence and deaths rely on the International epidemiology Databases to Evaluate AIDS (IeDEA) consortium for mortality estimates among children living with HIV (CHIV) receiving antiretroviral therapy (ART). Previous estimates, based on data through 2014, may no longer be accurate due to expanded paediatric HIV care and treatment eligibility, and the possibility of unreported deaths in CHIV considered lost to follow‐up (LTFU). We therefore estimated all‐cause mortality and its trends in CHIV (<15 years old) on ART using extended and new IeDEA data.

**Methods:**

We analysed (i) IeDEA observational data from CHIV in routine care globally, and (ii) novel data from an IeDEA tracing study that determined outcomes in a sample of CHIV after being LTFU in southern Africa. We included 45,711 CHIV on ART during 2004 to 2017 at 72 programmes in Africa, Asia‐Pacific and Latin America. We used mixed effects Poisson regression to estimate mortality by age, sex, CD4 at ART start, time on ART, region and calendar year. For Africa, in an adjusted analysis that accounts for unreported deaths among those LTFU, we first modified the routine data by simulating mortality outcomes within six months after LTFU, based on a Gompertz survival model fitted to the tracing data (*n* = 221).

**Results:**

Observed mortality rates were 1.8 (95% CI: 1.7 to 1.9) and 9.4 (6.3 to 13.4) deaths per 100 person‐years in the routine and tracing data, respectively. We found strong evidence of higher mortality at shorter ART durations, lower CD4 values, and in infancy. Averaging over covariate patterns, the adjusted mortality rate was 54% higher than the unadjusted rate. In unadjusted analyses, mortality reduced by an average 60% and 73% from 2005 to 2017, within and outside of Africa, respectively. In the adjusted analysis for Africa, this temporal reduction was 42%.

**Conclusions:**

Mortality rates among CHIV have decreased substantially over time. However, when accounting for worse outcomes among those LTFU, mortality estimates increased and temporal improvements were slightly reduced, suggesting caution in interpreting analyses based only on programme data. The improved and updated IeDEA estimates on mortality among CHIV on ART support UNAIDS efforts to accurately model global HIV statistics.

## 1 INTRODUCTION

Accurate estimates of the number of children living with HIV (CHIV) are essential for programme planning and resource allocation [1, 2]. Further, the number of deaths among CHIV and paediatric antiretroviral therapy (ART) coverage are important measures of programme effectiveness and progress towards targets such as the Joint United Nations Programme on HIV/AIDS (UNAIDS) 90‐90‐90 goal [3]. In many countries, these HIV statistics cannot be directly estimated, and we rely on mathematical models to derive them.

The UNAIDS Spectrum model (Spectrum) [4] generates robust global HIV estimates from country‐specific service statistics, survey and surveillance data, and epidemiological parameters derived from scientific studies. Previously, Spectrum incorporated estimates of mortality among CHIV on ART from the International epidemiology Databases to Evaluate AIDS (IeDEA) consortium using data through 2014 [5].

However, paediatric HIV programmes have evolved over time [6, 7, 8, 9]. Improved prevention of mother‐to‐child transmission, early infant diagnosis and PCR testing at birth, as well as earlier ART start and universal treatment, are likely to have impacted mortality, previously modelled in Spectrum as constant over time. Additionally, as the mortality estimates were derived from routine treatment programme data, and did not account for the possible under‐ascertainment of mortality in children recorded as lost to follow‐up (LTFU), mortality may have been underestimated [10].

In this study, we estimated all‐cause mortality rates and investigated their temporal trends among CHIV younger than 15 years on ART, during 2004 to 2017, in multiple regions within and outside of Africa. Unique to this analysis are the two distinct data sources: for all regions, we used routine‐care IeDEA observational data from participating treatment programmes; for southern Africa, we additionally used novel IeDEA data from a recent tracing study that determined outcomes in a sample of CHIV considered LTFU [11, 12]. The second data source provided insights into deaths that are potentially missed by programmes.

## 2 METHODS

### 2.1 Data sources

The IeDEA collaboration collects and harmonizes HIV/AIDS data globally [13, 14, 15, 16]. As of 2020, it had consolidated data on approximately two million people living with HIV from participating routine care treatment programmes, each comprised of multiple centres. In this analysis, we included all routine programmatic data (referred to as the “routine data,” below) on CHIV younger than 15 years, who were receiving ART (a combination of at least three antiretroviral drugs) initiated for the first time between 2001 and 2017. Six IeDEA regions contributed data: central Africa; East Africa; southern Africa; West Africa; Asia‐Pacific; and central America, South America and the Caribbean (hereafter ‘Latin America’).

We also utilized data from a recent IeDEA tracing study in six treatment programmes in five countries (Zambia, Malawi, Zimbabwe, Lesotho and Mozambique) in southern Africa. From the traced sample of 3256 persons classified as LTFU by participating programmes [11, 12], we used the subsample of CHIV aged younger than 15 years, who had not visited the clinic for at least the previous 120 to 180 days, depending on local definitions of LTFU. The children's outcomes were determined using phone calls and home visits. The sample was identified and traced during 2017 to 2019, with last clinic visits occurring during 2013 to 2017.

Within IeDEA [14], each participating programme has ethical approval from appropriate local institutions to collect and share patient data. Each region's data centre, which consolidates the data, has ethical approval to analyse the de‐identified data. At the time of ART initiation, CHIV or their caregivers provided consent for being traced.

### 2.2 Analysis

Analyses were performed in R version 3.6.3 [17] and Stata version 15.1 (Stata Corp., College Station, TX, USA). The analysis design was guided by Spectrum requirements [4, 18] and features of the data. We performed separate analyses for four distinct data subsets—separating the four sub‐Saharan African regions from the remaining two regions (Asia‐Pacific and Latin America); and, within each group of regions, separating children aged younger than five from those five to 14 years.

We performed two distinct analyses to produce: (i) “unadjusted” estimates of mortality, based on the routine data only; and (ii) for the African regions, “adjusted” estimates that incorporated the tracing study data. Secondary sensitivity analyses were also performed to investigate the impact of selected analysis decisions. All analyses are described below.

### 2.3 Unadjusted mortality rates

We analysed counts of deaths in the routine data using a multivariable Poisson mixed effects regression model (log link function). A child's time at risk started at the later of programme enrolment and ART initiation, and either ended at death (if recorded before age 15) or was censored at the earliest of turning 15 years old, being transferred to another programme, and last being seen alive, based on recorded clinic, laboratory and pharmacy visits. We excluded data beyond programme's centre‐specific “database closure dates”, from which we consider records incomplete.

Covariates included were consistent with how CHIV are distinguished in Spectrum [4]. These were time‐fixed sex and CD4 at ART initiation, and time‐varying time since ART initiation (<6 months, ≥6 months and <1 year, ≥1 year) and current age (<1, 1 to 2, 3 to 4, 5 to 9, 10 to 14 years). We included region as a covariate; and heterogeneity among programmes was captured by a normally distributed random effect.

To describe temporal trends in mortality, we included calendar year as a linear spline with a single knot in 2010 (Web Appendix E in Appendix S1 describes how the trend specification was chosen).

For the CD4 at ART initiation covariate, CD4 percentage measures were used in children younger than five years (<5, 5 to 10, 11 to 15, 16 to 20, 21 to 25, 26 to 30, >30%), and CD4 counts in CHIV aged at least five years (≤200, 200 to 349, 350 to 499, 500 to 749, 750 to 999, ≥1000 cells/mm^3^). From our IeDEA data, we extracted the CD4 measurement closest to, and within six months before and two weeks after, ART initiation. CD4 was modelled as pseudo‐continuous, using the (base 10) logarithm of the midpoint of the CD4 interval. In Spectrum [18], when CHIV turn five years old, CD4 percentages are translated into CD4 counts using a transition probability matrix [4]. Accordingly, we stochastically assigned CD4 count categories from a multinomial distribution using the transition probabilities (see Web Appendix A in Appendix S1) before analyzing the data. The simulation and analysis were repeated five times, and Rubin's rules [19] used to combine results. CHIV with missing CD4 values (44%) were excluded from the analysis, assuming that mortality outcomes are missing at random given model covariates.

Informed by likelihood ratio (LR) tests, we allowed for distinct covariate effects by ART duration (less than vs. at least one year), for each of CD4, region and the temporal trend.

### 2.4 Adjusted mortality rates

It is anticipated that a substantial number of child deaths remain unrecorded in the routine data because ART programmes in Africa do not generally link patient records to vital registration systems or use active follow‐up [20]. The adjusted mortality estimates attempt to account for these unrecorded deaths, using the southern Africa tracing data. The tracing data were used to adjust mortality for all sub‐Saharan African regions, assuming similarities in programmes and patterns of ascertainment of mortality in these regions, where most children receive care at public clinics following generally comparable national guidelines. We did not attempt to adjust estimates for Asia‐Pacific and Latin America, where children are more likely to receive care in specialized paediatric facilities and patterns of mortality ascertainment are likely to be different.

To generate adjusted mortality estimates, we applied an approach similar to that used for adult on‐ART mortality rate inputs for Spectrum [20] (see Web Appendix B in Appendix S1 for a graphic of our methods). To begin, for CHIV in the routine data who were considered LTFU by the treatment programmes, we simulated mortality outcomes over the six‐month period after becoming LTFU, based on a model determined from the tracing study data (the “simulation model” below). Thereafter, the analysis proceeded similarly to the unadjusted analysis above.

Consistent with the tracing study LTFU definitions, a child was considered LTFU (at the date last seen alive) if neither death nor transfer to another treatment programme were recorded, and the child did not access care within the six months preceding the centre database closure date. Simulation was restricted to six months after LTFU as it becomes increasingly uncertain whether a child remains on ART as this horizon is extended. The simulation and analysis were repeated 20 times, and results combined using Rubin's rules [19].

The simulation model was obtained by fitting a Gompertz proportional‐hazards survival model to the tracing study data, though restricting the number of parameters due to the small sample of 221 CHIV (see Web Appendix C in Appendix S1 for technical details on the simulation model and its application). Covariates included were sex, and, at the time of LTFU, time since ART initiation and age. In the tracing study, records at clinics were first examined and tracing only occurred if outcomes remained unknown—a binary indicator of whether tracing was required was also included. There was insufficient data to include CD4 at ART start. Calendar year of LTFU and time from LTFU to tracing were excluded based on LR tests. Programme was included as a gamma‐distributed random effect. Observations with missing values for covariates or outcomes (e.g., due to missing contact details) were excluded, assuming mortality outcomes are missing at random given model covariates (41% reduction in sample size).

The calendar years of LTFU in the routine data (2004 to 2017) were different from those in the tracing study (2013 to 2017). To reduce potential biases, the simulation model assumed that the temporal trends in mortality fitted to the unadjusted routine data also apply after LTFU (see Web Appendix C in Appendix S1).

### 2.5 Sensitivity analysis

We report the impact on estimated mortality of each of (i) simulating outcomes for 90 days or one year after LTFU, rather than six months; (ii) not including a time trend in the simulation model; (iii) moving all centre closure dates one year earlier (in case records are incomplete from earlier than indicated); and (iv) the multiple imputation of missing CD4 values.

## 3 RESULTS

### 3.1 Unadjusted analysis

Characteristics of the 45,711 CHIV in the routine data (after any data exclusions) are summarized in Table 1 (see Web Appendix D in Appendix S1 for disaggregation by region) and observed mortality rates in Table 2. Most of the CHIV were in African regions (92%), and primarily southern Africa (68%). Annual ART initiations peaked in 2009. The aggregate mortality rate was 1.8 deaths per 100 person‐years. Mortality rates were similar by sex, but varied by region, and were higher in children younger than five years and further in the past.

**Table 1:** Description of CHIV on ART in routine data

	Number of CHIV (%)		
	African regions: Central, East, Southern and West Africa	Other regions: Latin America and Asia‐Pacific	All regions
**Total**	41,836 (100.0)	3875 (100.0)	45,711 (100.0)
**Sex**			
Female	21,234 (50.8)	1878 (48.5)	23,112 (50.6)
Male	20,602 (49.2)	1997 (51.5)	22,599 (49.4)
**Region**			
Central Africa	839 (2.0)		839 (1.8)
East Africa	6982 (16.7)		6982 (15.3)
Southern Africa	31,012 (74.1)		31,012 (67.8)
West Africa	3003 (7.2)		3003 (6.6)
Asia‐Pacific		3285 (84.8)	3285 (7.2)
Latin America		590 (15.2)	590 (1.3)
**Age at ART start (whole years)**			
<1	5506 (13.2)	520 (13.4)	6026 (13.2)
1–2	7771 (18.6)	699 (18.0)	8470 (18.5)
3–4	4844 (11.6)	661 (17.1)	5505 (12.0)
5–9	13,535 (32.4)	1365 (35.2)	14,900 (32.6)
10–14	10,180 (24.3)	630 (16.3)	10,810 (23.6)
**CD4% at ART start, among ART start age <5 years**			
<5	971 (5.4)	329 (17.5)	1300 (6.5)
5–10	3147 (17.4)	321 (17.1)	3468 (17.3)
11–15	4107 (22.7)	341 (18.1)	4448 (22.2)
16–20	3678 (20.3)	338 (18.0)	4016 (20.1)
21–25	2483 (13.7)	210 (11.2)	2693 (13.5)
25–30	1537 (8.5)	148 (7.9)	1685 (8.4)
>30	2198 (12.1)	193 (10.3)	2391 (12.0)
**CD4 count at ART start (cells/mm^3^), among ART start age ≥5 years**			
<200	8708 (36.7)	1090 (54.6)	9798 (38.1)
200–349	5873 (24.8)	395 (19.8)	6268 (24.4)
350–499	3662 (15.4)	227 (11.4)	3889 (15.1)
500–749	2957 (12.5)	170 (8.5)	3127 (12.2)
750–999	1413 (6.0)	57 (2.9)	1470 (5.7)
≥1000	1102 (4.6)	56 (2.8)	1158 (4.5)
**Year of ART start** ^a^			
2001–2005	3612 (8.6)	229 (5.9)	3841 (8.4)
2006–2009	16,159 (38.6)	1880 (48.5)	18,039 (39.5)
2010–2012	12,863 (30.7)	1058 (27.3)	13,921 (30.5)
2013–2014	5511 (13.2)	449 (11.6)	5960 (13.0)
2015–2017	3691 (8.8)	259 (6.7)	3950 (8.6)

^a^ Boundaries of intervals chosen to correspond to ART eligibility guidelines changes. Abbreviations: ART, antiretroviral therapy; CHIV, children living with HIV.

**Table 2:** Observed mortality rates among CHIV on ART in routine data

			Mortality rate (deaths per 100 py)	
	Person‐years (py) at risk	Number of recorded deaths	Estimate	95% CI
**Total**	158,736	2871	1.81	1.74, 1.88
**Sex**				
Female	78,512	1370	1.74	1.65, 1.84
Male	80,223	1501	1.87	1.78, 1.97
**Region**				
Central Africa	3398	70	2.06	1.61, 2.60
East Africa	23,731	570	2.40	2.21, 2.61
Southern Africa	102,109	1554	1.52	1.45, 1.60
West Africa	11,028	380	3.45	3.11, 3.81
Asia‐Pacific	16,345	240	1.47	1.29, 1.67
Latin America	2126	57	2.68	2.03, 3.47
**Current age (whole years)**				
<5	37,306	1436	3.85	3.65, 4.05
5–14	121,430	1435	1.18	1.12, 1.24
**Current year** ^a^				
2001–2005	2394	233	9.73	8.52, 11.07
2006–2009	37,344	1217	3.26	3.08, 3.45
2010–2012	54,279	868	1.60	1.49, 1.71
2013–2014	35,360	380	1.07	0.97, 1.19
2015–2017	29,359	173	0.59	0.50, 0.68

^a^ Boundaries of intervals chosen to correspond to ART eligibility guidelines changes. Abbreviations: ART, antiretroviral therapy; CHIV, children living with HIV.

The fitted Poisson regression models are described in Table 3 (CHIV younger than five years), Table 4 (CHIV five to 14 years) and Table 5 (temporal trends). Associations between covariates and mortality were comparable in African and other regions. Within each region, there was substantial variation in mortality among programmes. In both age groups, there was strong evidence of lower mortality at longer ART durations (85% to 99% lower mortality when on ART for at least one year vs. less than six months) and higher CD4 at ART start (35% to 81% reduction in mortality from the lowest CD4 category to the next). Mortality was highest in infants (five‐ to six‐fold higher in children aged less than one year vs. three to four years). There was some evidence of regional differences within Africa. For CHIV younger than five years, mortality was lowest in southern Africa (even after accounting for this region's greater interprogramme variability). In older CHIV, regional differences varied more by ART duration, and central and West Africa experienced the highest mortality for shorter ART durations. From 2005 to each of 2010 and 2017, mortality rates decreased by 11% to 44% and 33% to 90%, respectively, depending on the group of regions, ages or ART durations (see Web Appendix E in Appendix S1 for the estimated model parameters). There was strong evidence that mortality declined from 2005 to 2017 for all groups, except among CHIV younger than five years on ART for at least one year.

**Table 3:** Mortality rate ratios and inter‐programme heterogeneity among CHIV on ART younger than five years, controlling for temporal trends, based on multivariable analysis of unadjusted routine data

	African regions				Latin America and Asia‐Pacific			
	ART duration <1 year		ART duration ≥1 year		ART duration <1 year		ART duration ≥1 year	
	Mortality rate ratio		Mortality rate ratio		Mortality rate ratio		Mortality rate ratio	
	Estimate (95% CI)	*p*‐Value	Estimate (95% CI)	*p*‐Value	Estimate (95% CI)	*p*‐Value	Estimate (95% CI)	*p*‐Value
**Sex** ^a^								
Male	Ref		Ref		Ref		Ref	
Female	0.94 (0.84, 1.05)	0.280	0.94 (0.84, 1.05)	0.280	0.94 (0.66, 1.32)	0.706	0.94 (0.66, 1.32)	0.706
**ART duration**								
<6 months	Ref		Ref		Ref		Ref	
≥6 months and <1 year	0.37 (0.31, 0.43)	<0.001			0.34 (0.21, 0.54)	<0.001		
≥1 year			0.15 (0.06, 0.36)	<0.001			0.04 (0.01, 0.29)	0.002
**Current age (whole years)** ^a^								
<1	5.67 (4.71, 6.82)	<0.001	5.67 (4.71, 6.82)	<0.001	4.79 (2.88, 7.99)	<0.001	4.79 (2.88, 7.99)	<0.001
1–2	2.24 (1.93, 2.60)	<0.001	2.24 (1.93, 2.60)	<0.001	1.75 (1.17, 2.61)	0.006	1.75 (1.17, 2.61)	0.006
3–4	Ref		Ref		Ref		Ref	
**CD4 % at ART initiation (%)** ^b^								
<5	Ref		Ref		Ref		Ref	
5–10	0.57 (0.52, 0.63)	<0.001	0.65 (0.51, 0.83)	<0.001	0.30 (0.24, 0.38)	<0.001	0.50 (0.25, 0.99)	0.047
11–15	0.44 (0.38, 0.51)		0.53 (0.37, 0.76)		0.17 (0.12, 0.24)		0.36 (0.13, 0.99)	
16–20	0.37 (0.31, 0.44)		0.47 (0.31, 0.71)		0.12 (0.08, 0.18)		0.29 (0.09, 0.99)	
21–25	0.33 (0.27, 0.40)		0.42 (0.26, 0.68)		0.09 (0.06, 0.15)		0.25 (0.06, 0.98)	
25–30	0.30 (0.24, 0.36)		0.39 (0.23, 0.66)		0.07 (0.04, 0.12)		0.22 (0.05, 0.98)	
>30	0.27 (0.22, 0.34)		0.37 (0.21, 0.64)		0.06 (0.04, 0.11)		0.20 (0.04, 0.98)	
**Region**								
Central Africa	0.64 (0.25, 1.63)	0.347	0.55 (0.14, 2.20)	0.403				
East Africa	Ref		Ref					
Southern Africa	0.56 (0.28, 1.13)	0.103	0.32 (0.15, 0.68)	0.003				
West Africa	1.18 (0.63, 2.19)	0.606	1.96 (0.61, 0.44)	0.934				
Asia‐Pacific					Ref		Ref	
Latin America					0.99 (0.24, 4.03)	0.986	1.10 (0.15, 8.26)	0.926

	Parameter estimate		Parameter estimate		Parameter estimate		Parameter estimate	
	Estimate (95% CI)	*p*‐Value	Estimate (95% CI)	*p*‐Value	Estimate (95% CI)	*p*‐Value	Estimate (95% CI)	*p*‐Value
**Random effect variance** ^a^ ^,^ ^c^								
All regions	0.10 (0.01, 0.91)	<0.001	0.10 (0.01, 0.91)	<0.001	0.66 (0.19, 2.27)	<0.001	0.66 (0.19, 2.27)	<0.001
Additional for Southern Africa	0.66 (0.25, 1.75)		0.66 (0.25, 1.75)					

^a^ Does not vary by ART duration by model design: the same estimates apply for ART <1 year and ART ≥1 year.

^b^ Included as pseudo‐continuous in the model, as a value of log_10_(2.5 + 5*i*) for the *i*th category.

^c^ Alternative interpretation of random effect variance: the 20% of programmes with the highest mortality have mortality rates at least 4.3, 1.7 and 3.9 times those in the 20% of programmes with the lowest mortality for southern Africa, other African regions, and Latin America/Asia‐Pacific, respectively. Abbreviations: ART, antiretroviral therapy; CHIV, children living with HIV.

**Table 4:** Mortality rate ratios and inter‐programme heterogeneity among CHIV on ART at least five years old, controlling for temporal trends, based on multivariable analysis of unadjusted routine data

	African regions				Latin America and Asia‐Pacific			
	ART duration <1 year		ART duration ≥1 year		ART duration <1 year		ART duration ≥1 year	
	Mortality rate ratio		Mortality rate ratio		Mortality rate ratio		Mortality rate ratio	
	Estimate (95% CI)	*p*‐Value	Estimate (95% CI)	*p*‐Value	Estimate (95% CI)	*p*‐Value	Estimate (95% CI)	*p*‐Value
**Sex** ^a^								
Male	Ref		Ref		Ref		Ref	
Female	0.97 (0.87, 1.08)	0.589	0.97 (0.87, 1.08)	0.589	0.87 (0.64, 1.19)	0.382	0.87 (0.64, 1.19)	0.38
**ART duration**								
<6 months	Ref		Ref		Ref		Ref	
≥6 months and <1 year	0.33 (0.27, 0.39)	<0.001			0.24 (0.14, 0.41)	<0.001		
≥1 year			0.07 (0.03, 0.19)	<0.001			0.00 (0.00, 0.05)	<0.001
**Current age (whole years)** ^a^								
5–9	Ref		Ref		Ref		Ref	
10–14	1.05 (0.94, 1.18)	0.393	1.05 (0.94, 1.18)	0.393	0.92 (0.65, 1.30)	0.632	0.92 (0.65, 1.30)	0.632
**CD4 count at ART initiation (cells/mm^3^)** ^b^								
<200	Ref		Ref		Ref		Ref	
200–349	0.43 (0.38, 0.48)	<0.001	0.54 (0.48, 0.61)	<0.001	0.19 (0.10, 0.35)	<0.001	0.53 (0.36, 0.78)	0.001
350–499	0.30 (0.25, 0.35)		0.42 (0.35, 0.49)		0.09 (0.04, 0.23)		0.40 (0.23, 0.70)	
500–749	0.22 (0.17, 0.27)		0.33 (0.27, 0.41)		0.05 (0.02, 0.15)		0.31 (0.16, 0.63)	
750–999	0.16 (0.13, 0.21)		0.27 (0.21, 0.34)		0.03 (0.01, 0.11)		0.25 (0.11, 0.58)	
≥1000	0.13 (0.10, 0.17)		0.23 (0.18, 0.30)		0.02 (0.00, 0.08)		0.22 (0.09, 0.55)	
**Region**								
Central Africa	2.03 (0.93, 4.41)	0.076	0.84 (0.36, 1.94)	0.677				
East Africa	Ref		Ref					
Southern Africa	1.08 (0.60, 1.95)	0.790	0.63 (0.35, 1.14)	0.126				
West Africa	3.13 (1.56, 6.28)	0.001	1.72 (0.85, 3.49)	0.130				
Asia‐Pacific					Ref		Ref	
Latin America					0.50 (0.15, 1.60)	0.242	1.57 (0.50, 4.97)	0.443

	Parameter estimate		Parameter estimate		Parameter estimate		Parameter estimate	
	Estimate (95% CI)	*p*‐Value	Estimate (95% CI)	*p*‐Value	Estimate (95% CI)	*p*‐Value	Estimate (95% CI)	*p*‐Value
Random effect variance^a^ ^,^ ^c^								
All regions	0.20 (0.04, 0.94)		0.20 (0.04, 0.94))		0.54 (0.19, 1.54)		0.54 (0.19, 1.54)	
Additional for Southern/West Africa	0.36 (0.09, 1.40)		0.36 (0.09, 1.40)					

^a^ Does not vary by ART duration by model design: the same estimates apply for ART <1 year and ART ≥1 year.

^b^ Included as pseudo‐continuous in the model, as the base 10 logarithm of either the midpoint of the interval or 1125 for the last category.

^c^ Alternative interpretation of random effect variance: the 20% of programmes with the highest mortality have mortality rates at least 3.5, 2.1 and 3.4 times those in the 20% of programmes with the lowest mortality, for southern/West Africa, other African regions, and Latin America/Asia‐Pacific, respectively. Abbreviations: ART, antiretroviral therapy; CHIV, children living with HIV.

**Table 5:** Mortality rate ratios describing temporal trends^a^ among CHIV on ART (reference year 2005), controlling for sex, age, ART duration, CD4 at ART start and region, based on multivariable analysis of unadjusted routine data

		African regions				Latin America and Asia‐Pacific			
		ART duration <1 year		ART duration ≥1 year		ART duration <1 year		ART duration ≥1 year	
Age group	Year	Estimate (95% CI)	*p*‐Value	Estimate (95% CI)	*p*‐Value	Estimate (95% CI)	*p*‐Value	Estimate (95% CI)	*p*‐Value
<5 years	2010	0.58 (0.48, 0.70)	<0.001	0.86 (0.45, 1.66)	0.655	0.61 (0.43, 0.88)	0.007	0.66 (0.22, 2.00)	0.463
	2017	0.21 (0.15, 0.30)	<0.001	0.67 (0.34, 1.29)	0.229	0.31 (0.13, 0.73)	0.007	0.37 (0.03, 5.27)	0.463
≥5 years	2010	0.62 (0.48, 0.79)	<0.001	0.66 (0.40, 1.09)	0.108	0.89 (0.43, 1.84)	0.758	0.56 (0.14, 2.21)	0.405
	2017	0.23 (0.16, 0.35)	<0.001	0.50 (0.31, 0.78)	0.002	0.30 (0.10, 0.91)	0.034	0.10 (0.03, 0.39)	<0.001

^a^ Calendar year is included in the regression model as a linear spline with knot at 2010. Abbreviations: ART, antiretroviral therapy; CHIV, children living with HIV.

### 3.2 Adjusted analysis

The 221 CHIV included in the tracing study data analysis, and their mortality rates, are described in Table 6. The median years from LTFU to tracing was 1.6 (quartile 1 to 3: 1.0 to 2.8). The aggregate mortality rate was 9.4 deaths per 100 person‐years (95% CI: 6.3 to 13.4), much higher than in the routine data and varying substantially by programme.

**Table 6:** Description of CHIV on ART described in tracing study data, and observed mortality rates

	Number of CHIV (%)	Number of deaths	Person‐years (py) at risk	Mortality rate per 100 py (95% CI)
**Total**	221 (100.0)	30	319	9.4 (6.3, 13.4)
**Sex**				
Female	120 (54.3)	11	174	6.3 (3.1, 11.3)
Male	101 (45.7)	19	144	13.2 (7.9, 20.5)
**Programme**				
1	9 (4.1)	0	14	0.0 (0.0, 26.2)
2	92 (41.6)	10	143	7.0 (3.4, 12.9)
3	19 (8.6)	2	28	7.2 (0.9, 26.0)
4	46 (20.8)	11	45	24.7 (12.3, 44.2)
5	29 (13.1)	2	51	3.9 (0.5, 14.2)
6	26 (11.8)	5	39	12.8 (4.2, 29.9)
**Age at LTFU (whole years)**				
<1	15 (6.8)	5	21	24.3 (7.9, 56.7)
1–2	58 (26.2)	9	86	10.5 (4.8, 19.9)
3–4	40 (18.1)	5	57	8.7 (2.8, 20.3)
5–14	108 (48.9)	11	155	7.1 (3.5, 12.7)
**ART duration at LTFU**				
<1 month^a^	29 (13.1)	8	42	18.9 (8.2, 37.3)
≥1 and <6 months	53 (24.0)	8	79	10.1 (4.4, 20.0)
≥6 months and <1 year	49 (22.2)	7	72	9.8 (3.9, 20.1)
≥1 year	90 (40.7)	7	126	5.5 (2.2, 11.4)
**Year of LTFU**				
2013	3 (1.4)	0	6	0.0 (0.0, 66.9)
2014	45 (20.4)	8	71	11.2 (4.8, 22.1)
2015	38 (17.2)	4	64	6.2 (1.7, 16.0)
2016	89 (40.3)	11	136	8.1 (4.0, 14.4)
2017	46 (20.8)	7	42	16.8 (6.7, 34.5)
**Time from LTFU to tracing (whole years)**				
0	49 (22.2)	6	43	13.8 (5.1, 30.0)
1	83 (37.6)	13	127	10.2 (5.4, 17.5)
2	39 (17.6)	3	67	4.4 (0.9, 13.0)
3 or 4	50 (22.6)	8	81	9.9 (4.3, 19.5)
**Tracing required**				
No	51 (23.1)	2	68	2.9 (0.4, 10.6)
Yes	170 (76.9)	28	251	11.2 (7.4, 16.1)

^a^ An additional ART duration category (compared to the routine data analysis) is included to account for stratified sampling in the tracing study. Abbreviations: ART, antiretroviral therapy; CHIV, children living with HIV.

The survival model fitted to the tracing study data (the basis of the simulation model, see Web Appendix C in Appendix S1) is presented in Table 7. Though uncertainties were large, higher mortality was associated with shorter ART durations (three‐fold higher for ART durations less than one month vs. greater than or equal to one year) and younger age (mortality was double in CHIV aged less than one year vs. greater than or equal to five years). Additionally, females experienced half the mortality rate experienced by males, and the need for tracing after examining records was associated with six‐fold higher mortality.

**Table 7:** Mortality rate ratios and model parameters for CHIV on ART after they are LTFU, based on multivariable analysis of tracing study data

	Mortality rate ratio	
	Estimate (95% CI)	*p*‐Value
**Sex**		
Male	Ref	
Female	0.47 (0.22, 1.04)	0.062
**ART duration**		
<1 month^a^	3.33 (1.04, 10.67)	0.042
≥1 and <6 months	1.31 (0.43, 3.99)	0.630
≥6 months and <1 year	1.34 (0.43, 4.16)	0.616
≥1 year	Ref	
**Current age (whole years)**		
<1	2.22 (0.68, 7.25)	0.188
1–2	1.22 (0.47, 3.18)	0.677
3–4	1.04 (0.35, 3.08)	0.943
5–14^b^	Ref	
**Tracing required**		
No	Ref	
Yes	6.14 (1.38, 27.25)	0.017

	Parameter estimate	
	Estimate (95% CI)	*p*‐Value
Random effect variance^c^, ^d^	0.38 (0.05, 2.63)	0.325
Gompertz ancillary parameter^c^	−1.84 (−2.76, −0.92)	<0.001

^a^ An additional ART duration category (compared to the routine data analysis) is included to account for stratified sampling in the tracing study

^b^ Categories are collapsed (compared to the routine data analysis) due to the small sample.

^c^ See Web Appendix B in Appendix S1 for the full model form specification.

^d^ Alternative interpretation of random effect variance: the 20% of programmes with the highest mortality have mortality rates at least 3.0 times those in the 20% of programmes with the lowest mortality. Abbreviations: ART, antiretroviral therapy; CHIV, children living with HIV.

For African regions, among the 42,898 CHIV who contributed data to the adjusted analysis, outcomes were simulated for the 25% identified as LTFU (see Web Appendix F in Appendix S1 for the proportions and characteristics of those LTFU). Those LFTU were equally split into males and females, and about a third (34%) were lost within six months of starting ART and two thirds (65%) were at least five years old.

**Figure 1:**
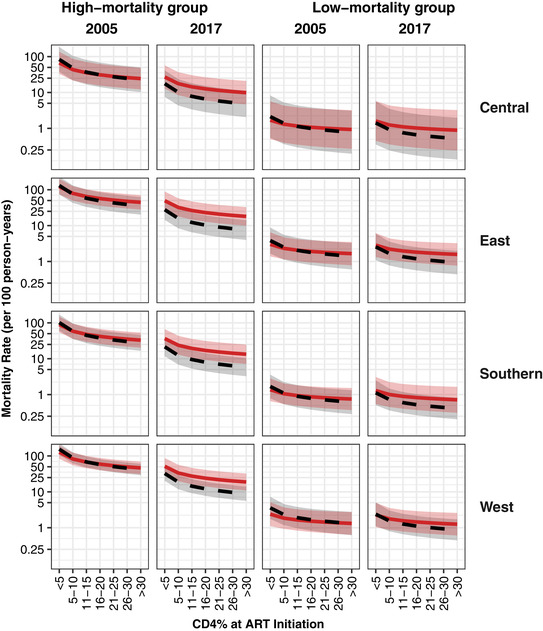
Model‐fitted mortality rates (and 95% CIs as shaded areas) among CHIV on ART younger than five years, both unadjusted using only the routine data (dashed black line) and adjusted by simulating outcomes for six months following LTFU (solid red line). Deaths per 100 person‐years (*y*‐axis; log scale) are shown by CD4% at ART start (*x*‐axis) for each of the four African regions (rows), for 2005 and 2017 (columns) and for high‐ and low‐mortality groups (columns) as defined by ART duration, age and sex (high: ART less than six months, age less than one year, male; low: ART greater than or equal to one year, age three to four years, female). Mortality rates are reported on a log scale to improve readability. ART, antiretroviral therapy; CHIV, children living with HIV.

**Figure 2:**
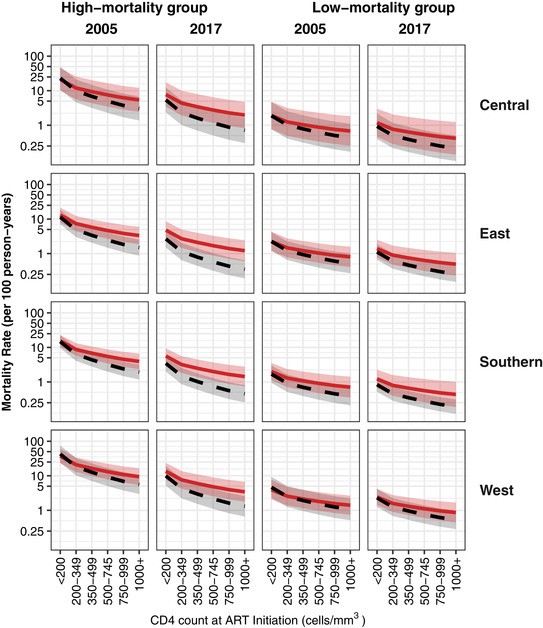
Model‐fitted mortality rates (and 95% CIs as shaded areas) among CHIV on ART at least five years old, both unadjusted when using only the routine data (dashed black line) and adjusted by simulating outcomes for six months following LTFU (solid red line). Deaths per 100 person‐years (*y*‐axis; log scale) are shown by CD4 count at ART start (*x*‐axis) for each of the four African regions (rows), for 2005 and 2017 (columns) and for high‐ and low‐mortality groups (columns) as defined by ART duration, age and sex (high: ART less than six months, age 10 to 14 years, male; low: ART greater than or equal to one year, age five to nine years, female). Mortality rates are reported on a log scale to improve readability. ART, antiretroviral therapy; CHIV, children living with HIV.

Unadjusted and adjusted mortality rates are compared in Figures 1 and 2 (CHIV younger than and at least five years old, respectively), by CD4 at ART start, for selected groups of the CHIV and years. Weighting each combination of covariate values and calendar year (2004 to 2017) equally, the adjusted mortality rate was on average 54% higher than the unadjusted rate (39% decrease to 4.3‐fold increase across covariate patterns). These relative increases were similar by region, but were larger in more recent years, at higher CD4 values, for the older age group, and for the high‐mortality groups (as defined by age, ART duration and sex—see Figures 1 and 2).

The associations between covariates and mortality were similar to those in the unadjusted analysis (see Web Appendices G and H in Appendix S1 for adjusted mortality rate ratios and a comparison of fitted mortality rates), though with some evidence of larger sex differences (females experienced 20% lower mortality than males), and an attenuated yet strong CD4 effect. Temporal trends were also reduced, though still substantial; mortality in 2017 was 5% to 64% lower than in 2005, depending on age group and ART duration.

### 3.3 Sensitivity analysis

The sensitivity analyses (see Web Appendix I in Appendix S1 for details) suggest that estimated adjusted mortality rates are generally stable, relative to uncertainties, against the decisions investigated about how to perform the adjustment for unreported deaths. Estimated mortality rates changed by at most 13%, averaging over covariate patterns. However, unadjusted results were more sensitive to the imputation of CD4 values—while the largest average change was of similar magnitude, there were some large changes for individual covariate patterns.

## 4 DISCUSSION

In this analysis of routine IeDEA programmatic data on more than 45,000 CHIV younger than 15 years on ART, we found that mortality rates substantially decreased over time. Mortality rates in 2017 were an average 60% and 73% lower than in 2005, in and outside of Africa, respectively, after controlling for individual characteristics, and with some variation by age, region and ART duration. However, our adjusted analysis, using novel IeDEA tracing study data [11, 12], suggests that analyses using only routine programme data may substantially underestimate mortality. The aggregate observed mortality rate in the southern Africa tracing study, which considered CHIV after LTFU, was five times greater than among CHIV recorded as in care by treatment programmes in the same region or globally (9.4 vs. 1.5 or 1.8 deaths per 100 person‐years, respectively). For African regions, the detailed analysis that adjusted for unreported deaths resulted in an average 54% increase in estimated mortality rates and a dampening of the temporal trend; mortality in 2017 was 42% lower than in 2005. These analyses produced mortality inputs for the UNAIDS Spectrum model that are significantly improved and updated, supporting more accurate modelling of global HIV statistics for the purposes of resource allocation.

Our study concurs with several previous analyses [21, 22, 23]: high mortality rates were associated with short ART durations, low CD4 values at ART start, and infancy. There was some variation by region, and, within each region, there was notable interprogramme variability, highlighting the need to incorporate this heterogeneity into mortality estimates while striving towards more equitable care. The temporal reductions in mortality estimates in our and other studies [24] suggest that guideline and programme implementation changes have improved child outcomes. This underscores the need to regularly update parameters used in HIV models.

It is recognized that retention in programmes remains a challenge in Africa [25, 26], thus, a key limitation to the accurate estimation of mortality rates from data collected by treatment programmes is the possibly different but unobserved mortality in those considered LTFU. Reasons for LTFU are diverse, including that some patients are in fact still participating in the same or another programme, but records do not accurately reflect this [11]. Importantly, some deaths may not be recorded in CHIV who are mistakenly considered LTFU [27, 28]. To our knowledge, the tracing data used in this analysis is from the first such study of more than 100 CHIV children on ART [29, 30], and the substantially higher mortality seen in children LTFU underscores the importance of conducting such studies to accurately assess outcomes and re‐engage individuals in care, efficient and effective methods of tracing, and improved record keeping to reduce misclassification as LTFU.

The relative adjustments in mortality rates, from incorporating the tracing data, are larger in more recent years and among older children. Some of the increasing adjustment with time may be explained by increasing rates of LTFU—the proportion of CHIV contributing data each calendar year, who are also LTFU in that year, grew from 4% in 2005 to 8% in 2016. While the factors associated with mortality in CHIV after LTFU were generally consistent with those found in the routine data, mortality after being LTFU was strongly associated with being male. This translated into higher mortality among males in the adjusted analysis of the routine data, highlighting that, as with older men, boys may be at greater risk of poorer outcomes [31, 32].

Many studies that estimate mortality from routine programme data treat LTFU as either non‐informative or use methods such as complete‐case analysis or multiple imputation, which often rely on assumptions leading to biases [10]. When outcomes are known in a subsample of those LTFU, determined through tracing studies or linkage to registries, inverse probability weighting can provide a practical approach to analyses [10]. However, in this analysis, we used a simulation approach [10, 20, 27] as outcomes were known for only a small sample of CHIV who were LFTU.

Limitations of this study include that IeDEA treatment programmes may not be representative of their region, though inclusion of multiple programmes and the use of random effects in analyses to describe heterogeneity mitigates this concern. Further limitations are the small sample (*n* = 221) of CHIV LTFU from the southern African region used to adjust mortality estimates for unreported deaths in all sub‐Saharan African regions, and the assumption that fitted temporal trends in mortality before LTFU hold after LTFU, incorporated without accounting for uncertainty. Other researchers have cautioned against generalizing programme‐specific results [30, 33]; and future updates of these mortality estimates would benefit from a larger dataset on outcomes in those LTFU, spanning additional years and regions. The large number of missing outcomes in the tracing study may have also introduced bias and highlights a remaining knowledge gap about those not successfully traced. Further studies within IeDEA are planned to determine outcomes either by tracing or linkage to death registries [33]; for example, the IeDEA central African region is initiating the first of its planned studies in Rwanda in 2021.

We did not attempt to adjust mortality estimates for regions outside of Africa due to the absence of tracing study data for these regions. While associations between covariates and mortality were comparable in African and other regions, we are limited in our ability to directly compare regions’ mortality rates. While it may be argued that the likelihood of unascertained mortality might be lower in Latin America and Asia‐Pacific regions [22, 34], we may be underestimating mortality by not applying any adjustments for potentially missed deaths in our analysis of these regions. Within African regions, by basing all adjustments on outcomes in a sample of those LTFU in southern Africa, which has relatively low mortality rates before LTFU, we may be under‐adjusting mortality in the other African regions. Even without these complexities, as we estimate all‐cause mortality in this study, many region‐specific factors not directly related to HIV could be contributing to any differences observed in mortality.

The absence of usable CD4 data in the tracing study data analysis, and thus omission of CD4 in the model used to simulate outcomes after LTFU, created caveats when interpreting results. For some covariate patterns, the adjustment for missed deaths resulted in a reduction of mortality, mainly at lower CD4 values; as CD4 could not be included in the simulation, we expect an under‐ and over‐estimation of adjusted mortality at smaller and larger CD4 values, respectively. The omission of CD4 is also likely to have increased the dampening of temporal trends, as the CD4 distribution has shifted upwards in recent years. The large amount of missing CD4 data in the routine data also posed challenges, as highlighted by the sensitivity of our results to alternative missingness assumptions. The increasing missingness of CD4 data raises questions about whether HIV models should be adapted to rely less on data no longer required to determine ART eligibility, or alternatively, whether the continued value of such data is being under‐emphasized for analysis and individual patient care purposes.

## 5 CONCLUSIONS

In this first analysis of mortality among CHIV on ART incorporating tracing data, we have demonstrated a substantial increase in mortality rate estimates for African regions upon adjusting for mortality in those classified as LTFU, which has important implications for interpreting reported mortality estimates from routine care settings. The substantial temporal improvements in mortality outcomes among CHIV on ART across our global cohorts are nevertheless reassuring, and highlight the need to further enhance efforts at ensuring early access to diagnosis and treatment, as well as retention in care, to optimize outcomes for CHIV.

## FUNDING

The IeDEA regions are supported by the National Institute on Drug Abuse (NIDA); the National Heart, Lung, and Blood Institute (NHLBI); the National Institute on Alcohol Abuse and Alcoholism (NIAAA); the National Institute of Diabetes and Digestive and Kidney Diseases (NIDDK); the Fogarty International Center (FIC); the Eunice Kennedy Shriver National Institute of Child Health and Human Development (NICHD); the National Cancer Institute (NCI); the National Institute of Allergy and Infectious Diseases (NIAID); the National Institute of Mental Health (NIMH); the Office of the Director, National Institutes of Health (OD); and the National Library of Medicine (NLM). The IeDEA regions are supported by grants U01AI069907 (TREAT Asia Pediatric HIV Observational Database), U01AI069923 (Caribbean, Central and South America network for HIV epidemiology), U01AI096299 (Central Africa), U01AI069911 (East Africa), U01AI069924 (Southern Africa) and U01AI069919 (West Africa). Informatics resources are supported by the Harmonist project (R24AI124872).

## DISCLAIMER

The content is solely the responsibility of the authors and does not necessarily represent the official views of the National Institutes of Health. Additional funding acknowledgements, site investigators and cohorts are listed in the Acknowledgements document in Appendix S2.

## COMPETING INTERESTS

The authors declare that they have no competing interests.

## AUTHORS’ CONTRIBUTIONS

MD, LFJ and CTY conceived the study. RK designed and performed the data analysis, with contributions by LFJ and MD RK drafted the article, with inputs from all authors. AE, TS, JP, DLD CT AK JGC and CK contributed to the routine data collection. EZ, LFJ and RK collated the routine data. MB and BC led the tracing studies and collated the tracing study data. All authors have read and approved the final version of the article.

## Supporting information

**Appendix S1**. Additional outputs and technical details

**Appendix S2**. Lists of additional funding acknowledgements, site investigators and cohorts, as referenced in Acknowledgements of the article.
