# Supplementary material for: Global HIV mortality trends among children on antiretroviral treatment corrected for under‐reported deaths: an updated analysis of the International epidemiology Databases to Evaluate AIDS collaboration
Source: J Int AIDS Soc. 2021 Sep 21;24(Suppl 5):e25780. doi: 10.1002/jia2.25780 (PMC8454681; doi:10.1002/jia2.25780)
Supplement: Supplementary file 1 — Appendix S1. Additional outputs and technical details [file JIA2-24-e25780-s002.pdf]

Web Appendix

This appendix provides supplementary content for the following article:

Reshma Kassanjee, Leigh F Johnson, Elizabeth Zaniewski, Marie Ballif, Benedikt Christ, Constantin T Yiannoutsos, Patience Nyakato, Sophie Desmonde, Andrew Edmonds, Tavitiya Sudjaritruk, Jorge Pinto, Rachel Vreeman, Désiré Lucien Dahourou, Christelle Twizere, Azar Kariminia, James G Carlucci, Charles Kasozi, Mary-Ann Davies, on behalf of the International Epidemiology Databases to Evaluate AIDS (IeDEA) Collaboration. Global HIV mortality trends among children on antiretroviral treatment corrected for under-reported deaths: An updated analysis of the International epidemiology Databases to Evaluate AIDS collaboration. *JIAS* 2021.

It is intended to be read in conjunction with the article, providing additional technical details or analysis outputs, as indicated in the main text.

Contents

Web Appendix A: Transition matrix for CD4% categories to CD4 count categories 2

Web Appendix B: Overview of analysis design 3

Web Appendix C: Simulation of outcomes in CHIV who are LTFU 4

Form of model fitted to the tracing study data 4

Simulation of outcomes from the fitted model 5

Web Appendix D: Description of CHIV on ART in the routine data analysis, by region 7

Web Appendix E: Fitted model coefficients for the calendar time terms, in the unadjusted routine data analysis 8

Web Appendix F: Proportions of CHIV who were LTFU and characteristics of LTFU CHIV in the adjusted routine data analysis 10

Web Appendix G: Unadjusted and adjusted estimated mortality rates, by covariate pattern 12

Web Appendix H: Fitted mortality rate ratios and model parameters, and implied temporal trends, in the adjusted analysis of African regions 17

Web Appendix I: Sensitivity Analyses 23

Adjustment of mortality rates to account for unreported deaths 23

Handling of missing CD4 data 33

Web Appendix A: Transition matrix for CD4% categories to CD4 count categories

Consistent with the Spectrum model structure[1], in our analysis of CHIV on ART, from age five onwards, CD4 *counts* at ART start are used to create distinct risk categories, instead of CD4 *percentages*.

The percentage of children moving into each CD4 count category, from each CD4% category, at age 5 years, as per Spectrum's design, is shown in Table A1 below.

Table A1: Transition matrix for translating CD4% categories to CD4 count categories at age five.

CD4%	n	Proportion in CD4 count (cells/mm^3) category (as a %)					
		[0,200)	[200,350)	[350,500)	[500,750)	[750,1000)	[1000,)
**[0,5)**	**695**	93	5	1	1	0	0
**[5,10]**	**1002**	34	37	17	10	2	0
**(10,15]**	**1174**	8	25	26	28	9	4
**(15,20]**	**819**	3	15	25	31	17	9
**(20,25]**	**482**	0	6	11	37	26	20
**(25,30]**	**305**	0	4	10	30	22	34
**(30,)**	**374**	1	2	6	11	19	61

[1] References: ● Stover J et al. Updates to the Spectrum/AIM model for estimating key HIV indicators at national and subnational levels. AIDS 2019. ● Stover J et al. Updates to the Spectrum model for the UNAIDS 2020 HIV estimates. JIAS 2021.

Web Appendix B: Overview of analysis design

The figure below complements the description of the analysis methods in the article. It shows the relationship between the data used for the unadjusted and adjusted analyses, and how the simulation model is used to modify the routine data for the adjusted analysis (see Web Appendix C for a detailed description of the simulation model structure and use). The data in the figure is artificial illustrative data.

Figure B1: Overview of analysis process

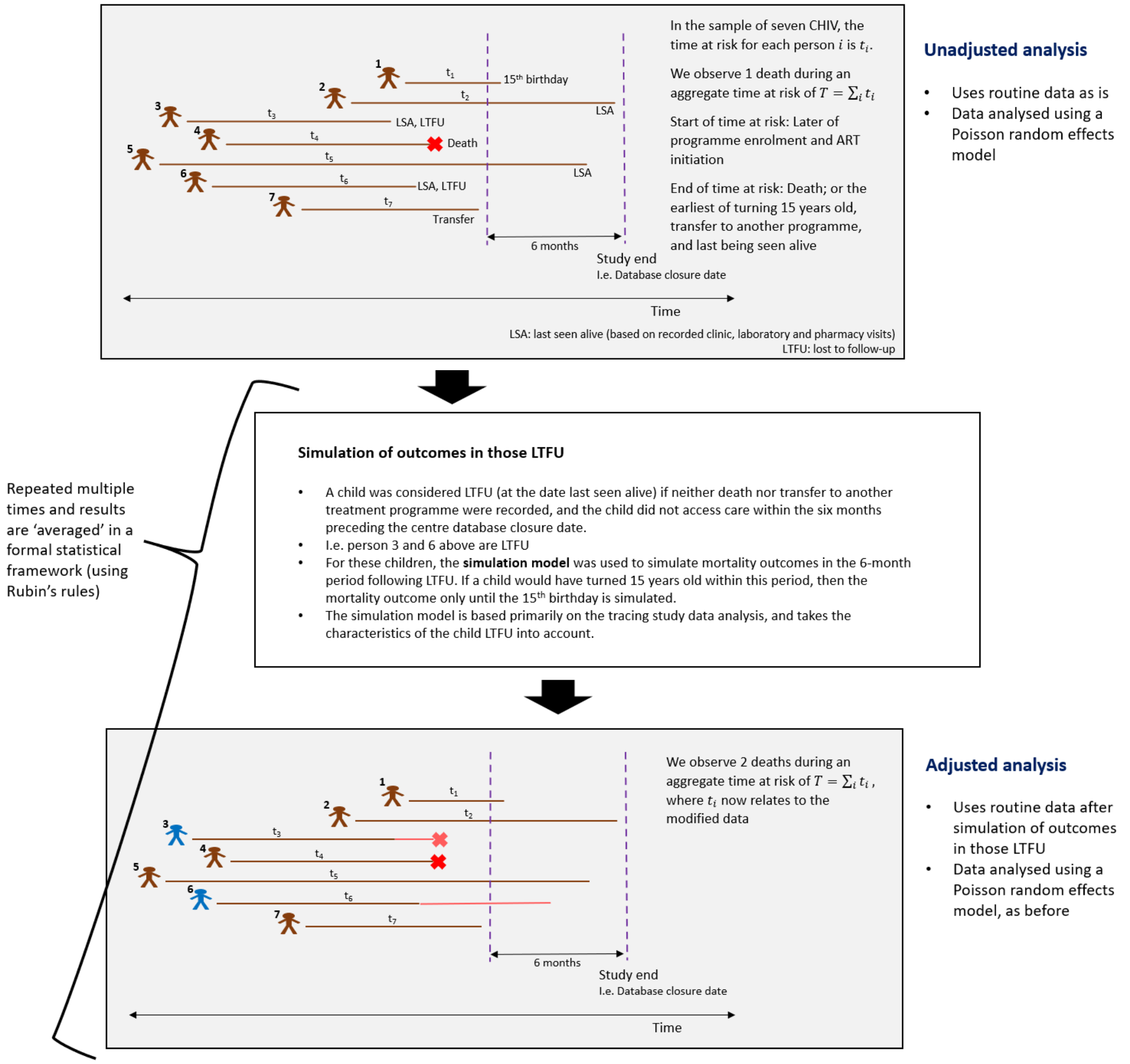

Web Appendix C: Simulation of outcomes in CHIV who are LTFU

The model obtained by analysis of the tracing study data, and the subsequent simulation of outcomes in those LTFU in the routine data, are each described below.

Form of model fitted to the tracing study data

Model selection

To select the form of the parametric proportional-hazards survival model fitted to the tracing study data, different distributions for the time from LTFU to death were compared, with the goal of obtaining the lowest AIC value (i.e., best 'goodness-of-fit' statistic) and, visually, good alignment between observed and model-fitted survival functions. Since the fitted model was going to be used to simulate outcomes in the routine data only for 6 months after LTFU, for each CHIV in the tracing study sample, data were censored at 2 years (6 months plus some margin) before model fitting. Of the distributions considered for the time from LTFU to death, namely Exponential, Weibull, Generalized Gamma and Gompertz distributions, the Gompertz distribution provided the best fit, and visually described the data well, over 2 years after LTFU.

A frailty term, i.e., a random effect, was also included to account for variation between programmes. The results were insensitive to the choice of distribution for the random effect when comparing Gamma and Inverse-gaussian distributions, and thus the Gamma distribution was used.

Final model specification

As per Stata's parameterization (see Stata documentation for the function `streg`), the specification of the fitted proportional-hazards model follows.

- The programme-specific random effects follow a $gamma\left(\frac{1}{\theta}, \theta\right)$ distribution, with mean 1 and variance θ, where θ is estimated during model fitting.

- For a CHIV with covariate values contained in the column vector $\underline{x}$:

  The marginal[2] probability of death *by* time t *after* LTFU is
  $$F_\theta(t) = 1 - \left(1 - \theta \ln\left(S(t)\right)\right)^{-\frac{1}{\theta}}$$
  where $S(t)$ is the survival function in the absence of random effects, and
  $$S(t) = \exp\left(-\frac{\lambda}{\gamma}(exp(\gamma t) - 1)\right)$$
  where $\lambda > 0$ and $\gamma \in \mathbb{R}$ are the parameters of the Gompertz distribution.

- The parameter γ is considered the ancillary parameter and directly estimated during model fitting. If $\gamma > 0$, the mortality rate $\lambda \cdot exp(\gamma t)$ increases exponentially with time after LTFU; if $\gamma = 0$, the rate is constant with time; and, if $\gamma < 0$, it decreases exponentially with time, as is the case in our setting.

- The parameter λ is related to the covariate values by $\lambda = \exp\left(\underline{x}^T \underline{\beta}\right)$, and the parameters contained in vector $\underline{\beta}$ are estimated.

---

[2] I.e., after accounting for the distribution of random effects which captures the inter-programme heterogeneity

Simulation of outcomes from the fitted model

Sampling from the distribution

For each CHIV in the routine data that was identified as LTFU, an outcome (whether death occurred, and, accordingly, the date of death) was simulated from the model described above, either (i) for 6 months after LTFU, or, (ii) until the child was no longer eligible to be included in the analysis, on the basis of age, if this occurred within the 6 months after LTFU. Given the characteristics of the CHIV and the estimated model parameters, the inverse probability integral transform was used to sample from the fitted distribution for time to death.

More specifically, for a CHIV with covariate values contained in the vector $\underline{x}$, and given model point estimates for $\lambda = \exp\,(\underline{x}^T\underline{\beta})$, γ and θ, denoted by $\hat{\lambda}$, $\hat{\gamma}$ and $\hat{\theta}$, respectively:

1. A probability p was sampled from a uniform distribution with support $[0,1]$.
2. The time from LTFU to death t was obtained by solving $F_\theta(t) = p$. This yields
$$t = \frac{1}{\hat{\gamma}} ln\left(1 - \frac{\hat{\gamma}}{\hat{\lambda}^*} ln\left(\exp\left(\frac{1-(1-p)^{-\hat{\theta}}}{\hat{\theta}}\right)\right)\right)$$
where $\hat{\lambda}^* = \hat{\lambda} \cdot f$ and the factor f accounts for a possible calendar time trend, further described below.
3. If $t > t_{max}$ the date of death is right censored at t_{max} after the LTFU date (outcome on this date is 'still alive'), otherwise the date of death is time t after the LTFU date. The value of t_{max} is the minimum of (i) 6 months, and (ii) the time from LTFU until the child would turn 15 years old.

Outcomes are generated in this way for all CHIV identified as LTFU in the routine data.

Combining the simulations

The process above was repeated to generate 20 distinct 'adjusted' datasets, and each dataset was analyzed to estimate mortality rates (see Methods of main article for a model description). The results from the 20 analyses were combined using Rubin's rules[3], assuming approximate normal distributions for the model parameters, when model parameters were reported, or for the logarithm of the estimated mortalities, when mortalities were reported.

To account for uncertainty in the simulation model, a bootstrapping approach was used. For each simulation, the tracing study model was fit to a random sample of the tracing study data (with replacement and maintaining the sample size), to obtain the point estimates $\hat{\lambda}$, $\hat{\gamma}$ and $\hat{\theta}$.

Covariates and time trends

The covariates included in the tracing study model are also required for each person LTFU in the routine data for the simulation, and are contained in $\underline{x}$ above. The covariates included in the tracing study model were sex (male versus female), age at LTFU (less than 1 year, 1-2 years, 3-4 years, or at least 5 years), ART duration at LTFU (less than 1 month, 1 month to less than 6 months, 6 months to less than 1 year, or at least 1 year) and whether tracing was required (yes or no). The last covariate, which describes whether active tracing (phones calls and visits) was required to determine outcomes, cannot be known for those CHIV identified as LTFU in the routine data. Therefore, the value of this covariate was also stochastically simulated for each CHIV LTFU, using probabilities of 'yes' and 'no' values consistent with their relative frequencies observed in the tracing study data (83.76% probability of 'yes').

---

[3] Reference: Rubin DB. Multiple imputation for nonresponse in surveys. New York: John Wiley & Sons, Inc; 1987.

To introduce a time trend, the time trends in mortality that were fitted to the unadjusted routine data (after controlling for covariates, as described in the main text) were assumed to also hold after LTFU (after controlling for the covariates in the tracing study model), with no uncertainty. More specifically:

- If the CHIV in the routine data who is LTFU was LTFU from 2014 to 2017, the simulation was performed as specified above with $f = 1$. That is, there was no modification of insights obtained from the tracing study data in the years for which that data were directly relevant.
- If the CHIV in the routine data who is LTFU was LTFU before 2014, suppose in year y, then the estimated λ obtained from the tracing study data was first modified by a factor f, equal to the mortality rate in year y relative to the mortality rate in 2015 (the median year of LTFU in the tracing study data). See Web Appendix E for the time trends fitted to the unadjusted routine data, used to calculate f, and Table 5 of the article for a summary of these time trends.

Web Appendix D: Description of CHIV on ART in the routine data analysis, by region

Table D1: Description of CHIV on ART in the routine data, by region

	Number of CHIV† (%)						
	**Southern Africa**	**East Africa**	**West Africa**	**Central Africa**	**Asia-Pacific**	**Latin America**	**All regions**
**Total**	31 012 (100.0)	6 982 (100.0)	3 003 (100.0)	839 (100.0)	3 285 (100.0)	590 (100.0)	45 711 (100.0)
**Sex**							
Female	15 832 (51.1)	3 549 (50.8)	1 434 (47.8)	419 (49.9)	1 566 (47.7)	312 (52.9)	23 112 (50.6)
Male	15 180 (48.9)	3 433 (49.2)	1 569 (52.2)	420 (50.1)	1 719 (52.3)	278 (47.1)	22 599 (49.4)
**Age at ART start (whole years)**							
<1	4 989 (16.1)	279 (4.0)	238 (7.9)	NA	439 (13.4)	81 (13.7)	6 026 (13.2)
1-2	5 986 (19.3)	947 (13.6)	666 (22.2)	172 (20.5)	622 (18.9)	77 (13.1)	8 470 (18.5)
3-4	3 391 (10.9)	899 (12.9)	420 (14.0)	134 (16.0)	603 (18.4)	58 (9.8)	5 505 (12.0)
5-9	9 307 (30.0)	2 875 (41.2)	1 097 (36.5)	256 (30.5)	1 167 (35.5)	198 (33.6)	14 900 (32.6)
10-14	7 339 (23.7)	1 982 (28.4)	582 (19.4)	277 (33.0)	454 (13.8)	176 (29.8)	10 810 (23.6)
**CD4% at ART start, among those starting ART < 5 years old (%)**							
<5	700 (4.9)	127 (6.0)	116 (8.8)	28 (9.2)	310 (18.6)	19 (8.8)	1 300 (6.5)
5-10	2 440 (17.0)	349 (16.4)	288 (21.8)	70 (22.9)	305 (18.3)	16 (7.4)	3 468 (17.3)
11-15	3 166 (22.0)	531 (25.0)	320 (24.2)	90 (29.4)	301 (18.1)	40 (18.5)	4 448 (22.2)
16-20	2 861 (19.9)	451 (21.2)	304 (23.0)	62 (20.3)	289 (17.4)	49 (22.7)	4 016 (20.1)
21-25	2 024 (14.1)	300 (14.1)	132 (10.0)	27 (8.8)	192 (11.5)	18 (8.3)	2 693 (13.5)
25-30	1 285 (8.9)	154 (7.2)	77 (5.8)	21 (6.9)	120 (7.2)	28 (13.0)	1 685 (8.4)
>30	1 890 (13.2)	213 (10.0)	87 (6.6)	8 (2.6)	147 (8.8)	46 (21.3)	2 391 (12.0)
**CD4 count at ART start, among those starting ART < 5 years old (cells/mm^3)**							
<200	6 067 (36.4)	1 695 (34.9)	750 (44.7)	196 (36.8)	928 (57.2)	162 (43.3)	9 798 (38.1)
200-349	4 238 (25.5)	1 153 (23.7)	357 (21.3)	125 (23.5)	323 (19.9)	72 (19.3)	6 268 (24.4)
350-499	2 659 (16.0)	698 (14.4)	228 (13.6)	77 (14.4)	163 (10.1)	64 (17.1)	3 889 (15.1)
500-749	2 049 (12.3)	644 (13.3)	197 (11.7)	67 (12.6)	121 (7.5)	49 (13.1)	3 127 (12.2)
750-999	913 (5.5)	375 (7.7)	90 (5.4)	35 (6.6)	42 (2.6)	15 (4.0)	1 470 (5.7)
≥1000	720 (4.3)	292 (6.0)	57 (3.4)	33 (6.2)	44 (2.7)	12 (3.2)	1 158 (4.5)
**Year of ART start‡**							
2001-2005	2 977 (9.6)	268 (3.8)	251 (8.4)	116 (13.8)	180 (5.5)	49 (8.3)	3 841 (8.4)
2006-2009	11 808 (38.1)	2 725 (39.0)	1 274 (42.4)	352 (42.0)	1 614 (49.1)	266 (45.1)	18 039 (39.5)
2010-2012	8 944 (28.8)	2 771 (39.7)	870 (29.0)	278 (33.1)	848 (25.8)	210 (35.6)	13 921 (30.5)
2013-2014	4 169 (13.4)	800 (11.5)	471 (15.7)	71 (8.5)	390 (11.9)	59 (10.0)	5 960 (13.0)
2015-2017	3 114 (10.0)	418 (6.0)	137 (4.6)	22 (2.6)	253 (7.7)	6 (1.0)	3 950 (8.6)

† As included in the final analysis, after any exclusion of observations during doing data preparation. ‡ Boundaries of intervals chosen to correspond to ART eligibility guidelines changes.

Web Appendix E: Fitted model coefficients for the calendar time terms, in the unadjusted routine data analysis

In the analysis of mortality rates, to describe temporal trends, we explored piecewise-constant, polynomial and linear spline forms of the calendar year term. We considered polynomials up to the fifth degree, and we considered including jumps in mortality for the piece-wise constant form or knots for the splines in the years of major treatment eligibility guideline changes – namely in 2010, 2013 and 2015. Forms were compared using the statistical criteria of AIC values and, when appropriate, likelihood-ratio (LR) tests, as well as visual assessment of the fitted trends – leading to the selection of a linear spline with a single knot in 2010.

The linear spline (with a knot at 2010) was used to relate the natural logarithm of mortality to calendar year. That is, there is a linear relationship between calendar year and the natural logarithm of the mortality rate, and the slope of the line changes at 2010. More specifically, the first and second basis functions $f_{t,1}(y)$ and $f_{t,2}(y)$ were included as covariates in the Poisson regression model,

where

- y is the current calendar year, and $y \in \{2004, 2005, \ldots, 2017\}$;
- $f_{t,1}(y) = \min\left\{\frac{y-2004}{13}, k\right\}$ and $f_{t,2}(y) = \max\left\{\frac{y-2004}{13}, k\right\} - k$; and
- $k = \frac{2010-2004}{13}$ specifies the placement of the knot.

This implies that, for a child with a given set of covariate values (region, sex, current age, current ART duration, CD4 at ART duration), the mortality rate in year y:

$$\lambda_y = \lambda_0 \cdot \exp\left(\beta_{t,1} \cdot f_{t,1}(y) + \beta_{t,2} \cdot f_{t,2}(y)\right)$$

where

- λ_0 is the mortality rate in 2004 for CHIV with the same covariate pattern, and
- $\beta_{t,1}$ and $\beta_{t,2}$ are model coefficients that are estimated during model fitting.

The estimated model coefficients, for each of the four subsets of data analysed (African versus other regions, and age <5 years versus 5-14 years), are provided in Table E1. The time trends among CHIV on ART for less than 1 year are distinct from those among CHIV on ART for at least 1 year. For the subset of data on CHIV outside of Africa and aged <5 years, the model form was simplified, as visually there appeared to be over-fitting to the relatively small mortality rates observed in this group, resulting in questionable fitted increases in mortality for periods. In the model for this subset of data

$$\lambda_y = \lambda_0 \cdot \exp\left(\beta_{t,o} \cdot f_{t,o}(y)\right)$$

where $f_{t,o}(y) = \frac{y-2004}{13}$ and $\beta_{t,o}$ is the model coefficient.

Table E1: Fitted model coefficients for calendar time terms in the unadjusted mortality analysis

	ART duration <1 year		ART duration ≥1 year	
	Estimate (95% CI)	p-value	Estimate (95% CI)	p-value
**Age <5 years, African regions**				
Spline basis function 1	-1.42 (-1.93,-0.92)	<0.001	-0.39 (-2.10,1.32)	0.655
Spline basis function 2	-1.87 (-2.56,-1.18)	<0.001	-0.48 (-1.64,0.69)	0.421
**Age <5 years, Asia-Pacific and Latin America**				
Linear term	-1.28 (-2.22,-0.34)	0.007	-1.08 (-3.96,1.80)	0.463
**Age 5-14 years, African regions**				
Spline basis function 1	-1.25 (-1.89,-0.61)	<0.001	-1.06 (-2.36,0.24)	0.108
Spline basis function 2	-1.81 (-2.62,-0.99)	<0.001	-0.55 (-1.16,0.07)	0.081
**Age 5-14 years, Asia-Pacific and Latin America**				
Spline basis function 1	-0.30 (-2.18,1.59)	0.758	-1.52 (-5.09,2.06)	0.405
Spline basis function 2	-2.01 (-4.36,0.33)	0.092	-3.13 (-5.13,-1.13)	0.002

Web Appendix F: Proportions of CHIV who were LTFU and characteristics of LTFU CHIV in the adjusted routine data analysis

For the African regions and the adjusted analysis, the proportions of CHIV who are LTFU are described in Table F1. The characteristics of these LTFU CHIV are described in Table F2.

Note that the total number of CHIV included in the adjusted analysis exceeds that in the unadjusted analysis (see Table 1 of main text for sample sizes) because the adjusted analysis also includes CHIV who are only observed once before becoming LTFU, while these CHIV cannot be used meaningfully in the unadjusted analysis. The 'percent LTFU' (Table F1) thus includes both those who are observed for some time in the routine database before becoming LTFU and those who become lost immediately (after contributing data for only one date, in the unadjusted routine data), while the 'percent immediately LTFU' measures the extent of only the latter. When disaggregating by age group or calendar year, the number of CHIV counts the CHIV who contribute any time at risk to the analysis for that age group or year, respectively, and the percent LTFU measures what percent of those CHIV is LTFU while the child is still in that age group or is LTFU during that same year.

Table F1: Percentage of total CHIV in the adjusted analysis who are LTFU

	Total number of CHIV[†]	Percent LTFU (number of CHIV)	Percent immediately LTFU (number of CHIV)
**Total**	42898	24.7 (10576)	2.5 (1066)
**Age (whole years)**			
<5	18744	20.0 (3741)	3.4 (629)
5-14	33690	20.3 (6835)	1.3 (437)
**Year**			
2004	878	2.2 (19)	0.7 (6)
2005	3557	4.2 (150)	1.5 (53)
2006	6505	4.5 (293)	0.6 (36)
2007	9312	5.2 (484)	0.9 (86)
2008	12444	5.5 (685)	0.9 (106)
2009	15970	5.5 (882)	0.9 (139)
2010	18819	5.0 (948)	0.6 (119)
2011	19937	5.5 (1087)	0.5 (106)
2012	20511	6.7 (1366)	0.5 (107)
2013	19884	5.8 (1156)	0.5 (95)
2014	17805	6.1 (1084)	0.4 (77)
2015	16935	6.8 (1150)	0.4 (76)
2016	13694	8.1 (1111)	0.3 (46)
2017[‡]	5521	2.9 (161)	0.3 (14)
**Region**			
Central	849	23.3 (198)	1.2 (10)
East	7111	22.7 (1617)	1.8 (129)
Southern	31821	25.7 (8188)	2.6 (813)
West	3117	18.4 (573)	3.7 (114)

[†] When split by age or year, the sum will exceed the total because a single CHIV may contribute data to more than one age group or year. [‡] The LTFU proportion is small as there is insufficient time at risk in 2017 to accurately observe who is (still) going to become LTFU in 2017.

Table F2: Characteristics of CHIV in the adjusted analysis who are LTFU

	Number of CHIV (%)
**Total**	10576 (100.0)
**Sex**	
Male	5197 (49.1)
Female	5379 (50.9)
**ART duration at LTFU**	
<1 month	1791 (16.9)
≥1 month and <6 months	1788 (16.9)
≥6 months and <1 year	1363 (12.9)
≥1 year	5634 (53.3)
**Age at LTFU (whole years)**	
<1	660 (6.2)
1-2	1758 (16.6)
3-4	1321 (12.5)
5-14	6837 (64.7)
**Region**	
Central	198 (1.9)
East	1617 (15.3)
Southern	8188 (77.4)
West	573 (5.4)
**Year of LTFU**	
2004	19 (0.2)
2005	150 (1.4)
2006	293 (2.8)
2007	484 (4.6)
2008	685 (6.5)
2009	882 (8.3)
2010	948 (9.0)
2011	1087 (10.3)
2012	1366 (12.9)
2013	1156 (10.9)
2014	1084 (10.3)
2015	1150 (10.9)
2016	1111 (10.5)
2017	161 (1.5)

Web Appendix G: Unadjusted and adjusted estimated mortality rates, by covariate pattern

The model-estimated mortality rates (deaths per 100 person-years), and 95% confidence intervals (CIs), are provided in Tables G1 and G2 (CHIV younger than 5 years, males and females in turn), and G3 and G4 (CHIV at least 5 years of age, males and female in turn). Both unadjusted and adjusted estimates are shown.

Due to the large number of results, mortality rates for only selected covariate patterns are shown. Mortality rates are shown by region and for each of three calendar years (2005, 2011, 2017), by every combination of the two most extreme categories for each of current age, CD4 at ART start, and ART duration. Note that children younger than 1 year cannot be on treatment on for at least 1 year, and therefore, in this age group, the longest ART duration is 6 months to 1 year.

Table G1: Unadjusted and adjusted model-estimated mortality rates per 100 person years (and 95% CIs) for male CHIV on ART younger than 5 years — by region and for calendar years 2005, 2011 and 2017; for combinations of the most extreme categories for current age (years), CD4% at ART initiation and ART duration (months/years)

Current Age	ART duration	CD4%	Year	Central Africa		East Africa		Southern Africa		West Africa		Asia-Pacific	Latin America
				Unadjusted	Adjusted	Unadjusted	Adjusted	Unadjusted	Adjusted	Unadjusted	Adjusted	Unadjusted	Unadjusted
**<1**	**<6m**	**<5**	**2005**	83.4 (37.7,184.1)	66.6 (32.9,134.7)	131.0 (74.2,231.3)	121.6 (74.8,197.7)	101.6 (60.8,169.7)	89.9 (54.5,148.2)	154.3 (107.0,222.3)	127.0 (83.5,193.1)	159.8 (74.0,345.0)	157.8 (36.3,687.0)
			**2011**	41.8 (19.0,91.9)	44.5 (22.2,89.1)	65.7 (37.5,115.1)	81.3 (50.7,130.4)	50.9 (30.6,84.9)	60.1 (36.7,98.4)	77.4 (54.1,110.7)	84.9 (56.4,127.8)	88.6 (42.4,185.0)	87.5 (20.3,377.2)
			**2017**	17.6 (7.5,41.3)	27.0 (12.8,56.9)	27.7 (14.4,53.1)	49.4 (27.5,88.7)	21.5 (11.8,39.2)	36.5 (19.9,66.9)	32.6 (20.3,52.4)	51.5 (30.4,87.3)	49.1 (19.4,124.5)	48.5 (10.0,234.1)
		**>30**	**2005**	22.7 (10.3,50.1)	24.4 (12.2,48.5)	35.6 (20.3,62.6)	44.5 (28.1,70.6)	27.6 (16.6,45.9)	32.9 (20.3,53.2)	42.0 (29.1,60.5)	46.4 (31.2,69.1)	9.9 (4.6,21.3)	9.7 (2.2,42.2)
			**2011**	11.4 (5.2,24.7)	16.3 (8.2,32.2)	17.9 (10.4,30.7)	29.8 (18.9,46.9)	13.9 (8.5,22.6)	22.0 (13.6,35.6)	21.0 (15.0,29.5)	31.1 (20.9,46.1)	5.5 (2.7,11.2)	5.4 (1.3,23.0)
			**2017**	4.8 (2.1,11.1)	9.9 (4.7,21.0)	7.5 (4.0,14.2)	18.1 (10.0,32.8)	5.8 (3.3,10.5)	13.3 (7.2,24.8)	8.9 (5.6,14.0)	18.9 (11.0,32.4)	3.0 (1.2,7.5)	3.0 (0.6,14.2)
	**6m-1y**	**<5**	**2005**	30.7 (13.7,69.2)	24.3 (11.3,52.1)	48.3 (26.7,87.5)	44.3 (25.5,77.2)	37.5 (21.9,64.2)	32.8 (18.8,57.2)	56.9 (38.0,85.1)	46.3 (28.1,76.2)	53.6 (21.5,133.6)	52.9 (11.2,249.5)
			**2011**	15.4 (6.9,34.4)	16.2 (7.7,34.4)	24.2 (13.5,43.4)	29.7 (17.3,50.9)	18.8 (11.0,32.0)	21.9 (12.7,37.9)	28.5 (19.3,42.1)	31.0 (19.0,50.4)	29.7 (12.5,70.6)	29.3 (6.4,135.4)
			**2017**	6.5 (2.7,15.4)	9.9 (4.4,21.9)	10.2 (5.2,19.9)	18.0 (9.5,34.2)	7.9 (4.3,14.7)	13.3 (6.9,25.5)	12.0 (7.3,19.8)	18.8 (10.4,33.9)	16.5 (5.9,45.5)	16.2 (3.2,82.7)
		**>30**	**2005**	8.4 (3.7,18.8)	8.9 (4.2,18.7)	13.1 (7.3,23.7)	16.2 (9.6,27.5)	10.2 (6.0,17.4)	12.0 (7.0,20.5)	15.5 (10.3,23.1)	16.9 (10.6,27.2)	3.3 (1.4,8.1)	3.3 (0.7,15.1)
			**2011**	4.2 (1.9,9.3)	5.9 (2.8,12.4)	6.6 (3.8,11.6)	10.8 (6.5,18.2)	5.1 (3.1,8.5)	8.0 (4.7,13.6)	7.8 (5.4,11.2)	11.3 (7.1,18.1)	1.8 (0.8,4.2)	1.8 (0.4,8.2)
			**2017**	1.8 (0.8,4.2)	3.6 (1.6,8.0)	2.8 (1.4,5.3)	6.6 (3.5,12.6)	2.2 (1.2,3.9)	4.9 (2.5,9.4)	3.3 (2.0,5.3)	6.9 (3.8,12.5)	1.0 (0.4,2.7)	1.0 (0.2,5.0)
**3-4**	**<6m**	**<5**	**2005**	14.7 (6.7,32.1)	14.3 (7.3,28.3)	23.1 (13.3,40.2)	26.2 (16.2,42.4)	17.9 (10.7,29.9)	19.3 (12.0,31.1)	27.2 (19.0,38.9)	27.3 (18.5,40.2)	33.3 (16.9,65.8)	32.9 (7.7,141.4)
			**2011**	7.4 (3.4,16.1)	9.6 (4.9,18.8)	11.6 (6.7,20.2)	17.5 (10.9,28.2)	9.0 (5.4,15.1)	12.9 (8.0,20.8)	13.6 (9.5,19.6)	18.3 (12.4,26.9)	18.5 (9.3,36.6)	18.3 (4.2,79.0)
			**2017**	3.1 (1.3,7.2)	5.8 (2.9,11.5)	4.9 (2.6,9.3)	10.6 (6.2,18.2)	3.8 (2.1,6.9)	7.8 (4.6,13.5)	5.8 (3.6,9.2)	11.1 (7.1,17.3)	10.2 (4.1,25.6)	10.1 (2.0,49.9)
		**>30**	**2005**	4.0 (1.8,8.8)	5.2 (2.6,10.4)	6.3 (3.6,11.0)	9.6 (5.9,15.6)	4.9 (2.9,8.2)	7.1 (4.3,11.5)	7.4 (5.1,10.7)	10.0 (6.7,14.9)	2.1 (0.9,4.7)	2.0 (0.4,9.3)
			**2011**	2.0 (0.9,4.4)	3.5 (1.8,7.0)	3.2 (1.8,5.4)	6.4 (3.9,10.4)	2.4 (1.5,4.1)	4.7 (2.9,7.8)	3.7 (2.6,5.3)	6.7 (4.4,10.1)	1.1 (0.5,2.6)	1.1 (0.2,5.2)
			**2017**	0.8 (0.4,2.0)	2.1 (1.0,4.3)	1.3 (0.7,2.5)	3.9 (2.2,6.9)	1.0 (0.6,1.9)	2.9 (1.6,5.1)	1.6 (1.0,2.5)	4.1 (2.5,6.7)	0.6 (0.2,1.7)	0.6 (0.1,3.2)
	**≥ 1y**	**<5**	**2005**	2.3 (0.6,8.7)	2.1 (0.6,6.8)	4.1 (1.8,9.4)	3.7 (1.8,7.7)	1.8 (0.8,3.8)	1.7 (0.8,3.3)	3.8 (1.9,7.7)	2.9 (1.4,6.0)	2.0 (0.4,9.7)	2.2 (0.2,26.7)
			**2011**	1.9 (0.5,6.8)	2.0 (0.7,6.4)	3.4 (1.7,6.8)	3.6 (2.1,6.3)	1.5 (0.8,2.8)	1.7 (0.9,3.0)	3.2 (1.8,5.6)	2.9 (1.7,5.0)	1.2 (0.4,3.9)	1.3 (0.1,12.5)
			**2017**	1.5 (0.4,6.0)	2.0 (0.6,6.8)	2.7 (1.2,6.3)	3.5 (1.7,7.2)	1.2 (0.5,2.6)	1.6 (0.7,3.6)	2.5 (1.2,5.4)	2.8 (1.3,5.8)	0.7 (0.1,5.1)	0.8 (0.1,12.1)
		**>30**	**2005**	0.8 (0.2,3.3)	1.1 (0.3,3.9)	1.5 (0.6,3.5)	2.0 (1.0,4.3)	0.7 (0.3,1.4)	0.9 (0.5,1.9)	1.4 (0.7,2.9)	1.6 (0.8,3.3)	0.4 (0.1,2.3)	0.4 (0.0,4.9)
			**2011**	0.7 (0.2,2.4)	1.1 (0.3,3.6)	1.2 (0.7,2.3)	2.0 (1.2,3.4)	0.5 (0.3,0.9)	0.9 (0.5,1.6)	1.2 (0.7,1.9)	1.6 (1.0,2.6)	0.2 (0.1,0.7)	0.3 (0.0,1.9)
			**2017**	0.6 (0.1,2.1)	1.1 (0.3,3.9)	1.0 (0.5,2.1)	1.9 (1.0,3.9)	0.4 (0.2,0.9)	0.9 (0.4,2.0)	0.9 (0.5,1.8)	1.5 (0.8,3.1)	0.1 (0.0,0.8)	0.2 (0.0,1.6)

m, months; y, years.

Table G2: Unadjusted and adjusted model-estimated mortality rates per 100 person years (and 95% CIs) for female CHIV on ART younger than 5 years — by region and for calendar years 2005, 2011 and 2017; for combinations of the most extreme categories for current age (years), CD4% at ART initiation and ART duration (years)

Current Age	ART duration	CD4%	Year	Central Africa		East Africa		Southern Africa		West Africa		Asia-Pacific	Latin America
				Unadjusted	Adjusted	Unadjusted	Adjusted	Unadjusted	Adjusted	Unadjusted	Adjusted	Unadjusted	Unadjusted
<1	<6m	<5	2005	78.5 (35.5,173.4)	54.5 (28.5,104.2)	123.3 (69.8,217.9)	99.5 (65.5,151.0)	95.7 (57.2,159.9)	73.5 (47.1,114.6)	145.3 (100.5,209.9)	103.8 (73.0,147.8)	149.5 (67.0,333.5)	147.6 (33.9,643.3)
			2011	39.4 (17.9,86.5)	36.4 (19.2,69.2)	61.8 (35.3,108.3)	66.5 (44.2,100.1)	48.0 (28.8,80.0)	49.2 (31.6,76.5)	72.8 (50.8,104.4)	69.5 (49.0,98.4)	82.9 (38.5,178.2)	81.9 (19.0,352.3)
			2017	16.6 (7.1,38.9)	22.1 (11.1,44.0)	26.1 (13.6,50.0)	40.4 (23.8,68.4)	20.2 (11.1,36.9)	29.8 (17.1,52.1)	30.7 (19.1,49.4)	42.2 (26.3,67.5)	45.9 (17.7,118.7)	45.3 (9.4,218.1)
		>30	2005	21.3 (9.7,47.2)	19.9 (10.6,37.6)	33.5 (19.1,58.9)	36.4 (24.5,54.1)	26.0 (15.7,43.2)	26.9 (17.5,41.3)	39.5 (27.3,57.1)	38.0 (27.2,53.1)	9.2 (4.2,20.0)	9.1 (2.1,39.0)
			2011	10.7 (4.9,23.3)	13.3 (7.1,25.1)	16.8 (9.8,28.9)	24.3 (16.4,36.1)	13.0 (8.0,21.3)	18.0 (11.6,27.8)	19.8 (14.1,27.8)	25.4 (18.1,35.7)	5.1 (2.5,10.5)	5.1 (1.2,21.2)
			2017	4.5 (1.9,10.5)	8.1 (4.0,16.3)	7.1 (3.8,13.4)	14.8 (8.6,25.4)	5.5 (3.1,9.8)	10.9 (6.1,19.4)	8.4 (5.3,13.2)	15.4 (9.4,25.3)	2.8 (1.1,7.0)	2.8 (0.6,13.1)
	6m-1y	<5	2005	28.9 (12.9,65.2)	19.9 (9.9,39.7)	45.5 (25.1,82.4)	36.3 (22.7,58.0)	35.3 (20.6,60.5)	26.8 (16.5,43.4)	53.6 (35.7,80.3)	37.9 (25.0,57.3)	50.1 (19.5,129.0)	49.5 (10.5,234.1)
			2011	14.5 (6.5,32.4)	13.3 (6.7,26.3)	22.8 (12.7,40.8)	24.3 (15.3,38.4)	17.7 (10.4,30.1)	17.9 (11.1,28.9)	26.9 (18.2,39.7)	25.3 (16.9,38.0)	27.8 (11.4,67.9)	27.4 (5.9,126.8)
			2017	6.1 (2.6,14.5)	8.1 (3.9,16.7)	9.6 (4.9,18.8)	14.7 (8.3,26.0)	7.5 (4.0,13.8)	10.9 (6.1,19.5)	11.3 (6.9,18.6)	15.4 (9.2,25.8)	15.4 (5.4,43.5)	15.2 (3.0,77.3)
		>30	2005	7.9 (3.5,17.7)	7.3 (3.7,14.3)	12.4 (6.9,22.3)	13.3 (8.5,20.6)	9.6 (5.6,16.4)	9.8 (6.2,15.5)	14.6 (9.7,21.8)	13.9 (9.4,20.4)	3.1 (1.3,7.6)	3.1 (0.7,14.0)
			2011	3.9 (1.8,8.7)	4.9 (2.5,9.5)	6.2 (3.5,10.9)	8.9 (5.7,13.8)	4.8 (2.9,8.0)	6.6 (4.1,10.4)	7.3 (5.0,10.6)	9.3 (6.3,13.7)	1.7 (0.7,4.0)	1.7 (0.4,7.5)
			2017	1.7 (0.7,3.9)	2.9 (1.4,6.2)	2.6 (1.4,5.0)	5.4 (3.0,9.6)	2.0 (1.1,3.7)	4.0 (2.2,7.2)	3.1 (1.9,5.0)	5.6 (3.3,9.6)	0.9 (0.4,2.5)	0.9 (0.2,4.6)
3-4	<6m	<5	2005	13.8 (6.3,30.3)	11.7 (5.6,24.3)	21.8 (12.5,37.9)	21.4 (12.2,37.6)	16.9 (10.1,28.2)	15.8 (9.0,27.9)	25.6 (17.9,36.8)	22.3 (13.6,36.7)	31.2 (15.5,62.7)	30.8 (7.2,131.1)
			2011	6.9 (3.2,15.2)	7.8 (3.8,16.3)	10.9 (6.3,19.0)	14.3 (8.1,25.1)	8.5 (5.0,14.2)	10.6 (6.0,18.7)	12.9 (8.9,18.5)	14.9 (9.1,24.6)	17.3 (8.6,34.6)	17.1 (4.0,73.1)
			2017	2.9 (1.3,6.8)	4.8 (2.3,9.9)	4.6 (2.4,8.8)	8.7 (4.7,16.0)	3.6 (1.9,6.5)	6.4 (3.5,11.9)	5.4 (3.4,8.7)	9.1 (5.3,15.6)	9.6 (3.8,24.1)	9.5 (1.9,46.1)
		>30	2005	3.8 (1.7,8.3)	4.3 (2.0,9.0)	5.9 (3.4,10.3)	7.8 (4.4,13.9)	4.6 (2.7,7.7)	5.8 (3.2,10.3)	7.0 (4.8,10.1)	8.2 (4.9,13.6)	1.9 (0.9,4.3)	1.9 (0.4,8.5)
			2011	1.9 (0.9,4.1)	2.9 (1.4,6.0)	3.0 (1.7,5.1)	5.2 (2.9,9.3)	2.3 (1.4,3.8)	3.9 (2.1,7.0)	3.5 (2.4,5.0)	5.5 (3.2,9.2)	1.1 (0.5,2.4)	1.1 (0.2,4.7)
			2017	0.8 (0.3,1.8)	1.7 (0.8,3.7)	1.3 (0.7,2.4)	3.2 (1.7,6.1)	1.0 (0.5,1.8)	2.3 (1.2,4.5)	1.5 (0.9,2.4)	3.3 (1.8,6.0)	0.6 (0.2,1.6)	0.6 (0.1,2.9)
	≥ 1y	<5	2005	2.1 (0.5,8.2)	1.7 (0.5,5.7)	3.8 (1.7,8.8)	3.0 (1.4,6.5)	1.7 (0.8,3.6)	1.4 (0.7,2.8)	3.6 (1.8,7.3)	2.4 (1.1,5.1)	1.8 (0.4,9.2)	2.0 (0.2,25.0)
			2011	1.8 (0.5,6.4)	1.7 (0.5,5.3)	3.2 (1.6,6.4)	3.0 (1.6,5.4)	1.4 (0.7,2.7)	1.4 (0.7,2.6)	3.0 (1.7,5.3)	2.4 (1.3,4.3)	1.1 (0.3,3.7)	1.2 (0.1,11.7)
			2017	1.4 (0.4,5.6)	1.6 (0.4,5.7)	2.6 (1.1,6.0)	2.9 (1.3,6.1)	1.1 (0.5,2.5)	1.3 (0.5,3.1)	2.4 (1.1,5.1)	2.3 (1.0,5.0)	0.7 (0.1,4.8)	0.7 (0.0,11.3)
		>30	2005	0.8 (0.2,3.1)	0.9 (0.3,3.2)	1.4 (0.6,3.3)	1.7 (0.8,3.5)	0.6 (0.3,1.3)	0.8 (0.4,1.5)	1.3 (0.6,2.8)	1.3 (0.6,2.7)	0.4 (0.1,2.1)	0.4 (0.0,4.5)
			2011	0.6 (0.2,2.3)	0.9 (0.3,3.0)	1.2 (0.6,2.2)	1.6 (1.0,2.7)	0.5 (0.3,0.9)	0.7 (0.4,1.3)	1.1 (0.7,1.8)	1.3 (0.8,2.2)	0.2 (0.1,0.7)	0.2 (0.0,1.7)
			2017	0.5 (0.1,2.0)	0.9 (0.2,3.2)	0.9 (0.4,2.0)	1.6 (0.8,3.2)	0.4 (0.2,0.8)	0.7 (0.3,1.6)	0.9 (0.4,1.7)	1.3 (0.6,2.6)	0.1 (0.0,0.7)	0.1 (0.0,1.5)

m, months; y, years.

Table G3: Unadjusted and adjusted model-estimated mortality rates per 100 person years (and 95% CIs) for male CHIV on ART at least 5 years old — by region and for calendar years 2005, 2011 and 2017; for combinations of the most extreme categories for current age (years), CD4 count at ART initiation (cells/mm^3) and ART duration (months/years)

Current Age	ART duration	CD4 count	Year	Central Africa		East Africa		Southern Africa		West Africa		Asia-Pacific	Latin America
				Unadjusted	Adjusted	Unadjusted	Adjusted	Unadjusted	Adjusted	Unadjusted	Adjusted	Unadjusted	Unadjusted
**5-9**	**<6m**	**<200**	**2005**	21.9 (10.2,46.9)	21.0 (9.9,44.5)	10.8 (6.7,17.4)	12.9 (8.0,20.9)	14.0 (9.2,21.4)	15.2 (10.5,22.2)	40.5 (23.0,71.1)	36.5 (23.1,57.8)	17.8 (9.3,33.8)	8.8 (2.7,29.5)
			**2011**	11.8 (5.6,25.1)	12.6 (5.9,26.6)	5.8 (3.7,9.3)	7.7 (4.8,12.4)	7.6 (5.0,11.4)	9.1 (6.3,13.3)	21.8 (12.5,38.0)	21.9 (13.9,34.4)	13.6 (7.7,24.0)	6.8 (2.0,22.4)
			**2017**	5.1 (2.2,11.8)	7.6 (3.4,17.3)	2.5 (1.4,4.6)	4.7 (2.6,8.4)	3.3 (1.9,5.7)	5.6 (3.3,9.3)	9.5 (4.9,18.2)	13.3 (7.7,23.1)	5.4 (1.7,17.0)	2.7 (0.5,13.1)
		**≥1000**	**2005**	3.6 (1.6,7.8)	6.2 (2.8,13.5)	1.8 (1.0,2.9)	3.8 (2.2,6.6)	2.3 (1.4,3.7)	4.5 (2.7,7.3)	6.6 (3.6,12.0)	10.7 (6.3,18.2)	0.5 (0.1,2.1)	0.2 (0.0,1.4)
			**2011**	1.9 (0.9,4.1)	3.7 (1.7,8.1)	0.9 (0.6,1.5)	2.3 (1.3,3.9)	1.2 (0.8,1.9)	2.7 (1.6,4.4)	3.5 (2.0,6.4)	6.4 (3.8,10.8)	0.4 (0.1,1.5)	0.2 (0.0,1.0)
			**2017**	0.8 (0.4,1.9)	2.2 (0.9,5.4)	0.4 (0.2,0.7)	1.4 (0.7,2.7)	0.5 (0.3,0.9)	1.6 (0.9,3.1)	1.5 (0.8,3.0)	3.9 (2.1,7.5)	0.1 (0.0,0.8)	0.1 (0.0,0.5)
	**≥ 1 y**	**<200**	**2005**	1.9 (0.8,4.8)	2.4 (0.9,5.9)	2.3 (1.2,4.3)	2.8 (1.5,5.2)	1.7 (1.0,3.1)	2.5 (1.4,4.4)	4.8 (2.4,9.6)	4.9 (2.6,9.2)	2.3 (0.7,7.8)	3.6 (0.8,16.6)
			**2011**	1.2 (0.5,2.8)	1.4 (0.6,3.2)	1.5 (0.9,2.4)	1.6 (1.0,2.6)	1.1 (0.7,1.7)	1.5 (0.9,2.2)	3.0 (1.7,5.3)	2.9 (1.8,4.5)	1.0 (0.5,1.8)	1.6 (0.5,5.0)
			**2017**	1.0 (0.4,2.2)	1.4 (0.6,3.8)	1.1 (0.7,1.9)	1.7 (0.9,3.1)	0.9 (0.5,1.4)	1.5 (0.8,2.8)	2.4 (1.3,4.3)	3.0 (1.7,5.1)	0.2 (0.1,0.6)	0.4 (0.1,1.6)
		**≥1000**	**2005**	0.5 (0.2,1.3)	0.9 (0.3,2.6)	0.6 (0.3,1.2)	1.1 (0.5,2.2)	0.5 (0.3,0.9)	1.0 (0.5,2.0)	1.3 (0.6,2.6)	1.9 (1.0,3.7)	0.6 (0.1,2.5)	0.9 (0.2,4.9)
			**2011**	0.3 (0.1,0.8)	0.6 (0.2,1.4)	0.4 (0.2,0.6)	0.6 (0.4,1.2)	0.3 (0.2,0.5)	0.6 (0.3,1.1)	0.8 (0.5,1.5)	1.1 (0.7,1.9)	0.3 (0.1,0.6)	0.4 (0.1,1.4)
			**2017**	0.3 (0.1,0.6)	0.6 (0.2,1.7)	0.3 (0.2,0.5)	0.7 (0.3,1.5)	0.2 (0.1,0.4)	0.6 (0.2,1.4)	0.6 (0.3,1.2)	1.2 (0.6,2.4)	0.1 (0.0,0.2)	0.1 (0.0,0.4)
**10-14**	**<6m**	**<200**	**2005**	23.0 (10.8,49.1)	21.2 (10.0,45.2)	11.4 (7.1,18.2)	13.1 (8.0,21.3)	14.7 (9.6,22.6)	15.4 (10.5,22.7)	42.5 (24.1,75.0)	37.0 (23.0,59.5)	16.3 (8.1,32.8)	8.1 (2.4,27.1)
			**2011**	12.4 (5.9,26.2)	12.7 (6.0,27.0)	6.1 (3.9,9.7)	7.8 (4.9,12.6)	7.9 (5.3,12.0)	9.3 (6.3,13.6)	22.9 (13.2,40.0)	22.2 (14.0,35.3)	12.5 (6.9,22.8)	6.2 (1.9,20.3)
			**2017**	5.4 (2.4,12.4)	7.7 (3.4,17.4)	2.7 (1.5,4.8)	4.8 (2.7,8.5)	3.4 (2.0,5.9)	5.6 (3.4,9.4)	10.0 (5.2,19.1)	13.5 (7.7,23.5)	4.9 (1.5,15.8)	2.5 (0.5,11.8)
		**≥1000**	**2005**	3.7 (1.7,8.2)	6.2 (2.8,13.7)	1.8 (1.1,3.1)	3.8 (2.2,6.8)	2.4 (1.5,3.9)	4.5 (2.8,7.5)	6.9 (3.7,12.7)	10.9 (6.3,18.7)	0.5 (0.1,2.0)	0.2 (0.0,1.3)
			**2011**	2.0 (0.9,4.3)	3.7 (1.7,8.2)	1.0 (0.6,1.6)	2.3 (1.3,4.0)	1.3 (0.8,2.0)	2.7 (1.7,4.4)	3.7 (2.1,6.7)	6.5 (3.8,11.1)	0.3 (0.1,1.4)	0.2 (0.0,1.0)
			**2017**	0.9 (0.4,2.0)	2.3 (1.0,5.4)	0.4 (0.2,0.8)	1.4 (0.7,2.8)	0.6 (0.3,1.0)	1.7 (0.9,3.1)	1.6 (0.8,3.2)	4.0 (2.1,7.6)	0.1 (0.0,0.8)	0.1 (0.0,0.5)
	**≥ 1 y**	**<200**	**2005**	2.0 (0.8,5.0)	2.4 (1.0,6.0)	2.4 (1.3,4.5)	2.8 (1.5,5.3)	1.8 (1.0,3.3)	2.5 (1.4,4.4)	5.0 (2.5,10.1)	4.9 (2.6,9.4)	2.1 (0.6,7.3)	3.3 (0.7,15.3)
			**2011**	1.3 (0.6,2.9)	1.4 (0.6,3.2)	1.6 (1.0,2.5)	1.7 (1.0,2.7)	1.2 (0.8,1.8)	1.5 (1.0,2.3)	3.2 (1.8,5.6)	2.9 (1.9,4.6)	0.9 (0.5,1.7)	1.4 (0.5,4.6)
			**2017**	1.0 (0.4,2.3)	1.5 (0.6,3.8)	1.2 (0.7,2.0)	1.7 (1.0,3.1)	0.9 (0.6,1.4)	1.5 (0.8,2.8)	2.5 (1.4,4.5)	3.0 (1.8,5.1)	0.2 (0.1,0.6)	0.3 (0.1,1.4)
		**≥1000**	**2005**	0.5 (0.2,1.4)	0.9 (0.3,2.6)	0.7 (0.3,1.3)	1.1 (0.5,2.3)	0.5 (0.3,0.9)	1.0 (0.5,2.0)	1.3 (0.6,2.8)	1.9 (1.0,3.8)	0.5 (0.1,2.4)	0.8 (0.1,4.6)
			**2011**	0.3 (0.2,0.8)	0.6 (0.2,1.5)	0.4 (0.3,0.7)	0.7 (0.4,1.2)	0.3 (0.2,0.5)	0.6 (0.3,1.1)	0.9 (0.5,1.5)	1.2 (0.7,2.0)	0.2 (0.1,0.6)	0.4 (0.1,1.3)
			**2017**	0.3 (0.1,0.6)	0.6 (0.2,1.7)	0.3 (0.2,0.5)	0.7 (0.3,1.5)	0.2 (0.2,0.4)	0.6 (0.3,1.4)	0.7 (0.4,1.2)	1.2 (0.6,2.4)	0.1 (0.0,0.2)	0.1 (0.0,0.4)

m, months; y, years.

Table G4: Unadjusted and adjusted model-estimated mortality rates per 100 person years (and 95% CIs) for female CHIV on ART at least 5 years old — by region and for calendar years 2005, 2011 and 2017; for combinations of the most extreme categories for current age (years), CD4 count at ART initiation (cells/mm^3) and ART duration (months/years)

Current Age	ART duration	CD4 count	Year	Central Africa		East Africa		Southern Africa		West Africa		Asia-Pacific	Latin America
				Unadjusted	Adjusted	Unadjusted	Adjusted	Unadjusted	Adjusted	Unadjusted	Adjusted	Unadjusted	Unadjusted
**5-9**	**<6m**	**<200**	**2005**	21.2 (9.9,45.5)	17.1 (8.0,36.3)	10.5 (6.5,16.9)	10.5 (6.5,17.1)	13.6 (8.9,20.8)	12.4 (8.3,18.5)	39.3 (22.3,69.0)	29.8 (18.8,47.2)	15.4 (8.1,29.6)	7.7 (2.3,25.7)
			**2011**	11.5 (5.4,24.4)	10.2 (4.8,21.9)	5.7 (3.6,9.0)	6.3 (3.8,10.3)	7.3 (4.8,11.1)	7.4 (4.9,11.3)	21.2 (12.2,36.8)	17.8 (11.2,28.5)	11.8 (6.7,21.0)	5.9 (1.8,19.5)
			**2017**	5.0 (2.2,11.5)	6.2 (2.7,14.1)	2.5 (1.4,4.4)	3.8 (2.1,6.9)	3.2 (1.8,5.5)	4.5 (2.6,7.8)	9.2 (4.8,17.7)	10.9 (6.2,19.0)	4.7 (1.5,15.0)	2.3 (0.5,11.5)
		**≥1000**	**2005**	3.4 (1.6,7.6)	5.0 (2.3,11.1)	1.7 (1.0,2.9)	3.1 (1.8,5.4)	2.2 (1.4,3.5)	3.6 (2.2,6.1)	6.4 (3.5,11.7)	8.7 (5.1,14.9)	0.4 (0.1,1.8)	0.2 (0.0,1.2)
			**2011**	1.9 (0.9,4.0)	3.0 (1.4,6.7)	0.9 (0.6,1.5)	1.8 (1.0,3.3)	1.2 (0.8,1.9)	2.2 (1.3,3.7)	3.4 (1.9,6.2)	5.2 (3.1,9.0)	0.3 (0.1,1.3)	0.2 (0.0,0.9)
			**2017**	0.8 (0.3,1.9)	1.8 (0.8,4.4)	0.4 (0.2,0.7)	1.1 (0.6,2.3)	0.5 (0.3,0.9)	1.3 (0.7,2.6)	1.5 (0.8,2.9)	3.2 (1.7,6.1)	0.1 (0.0,0.7)	0.1 (0.0,0.5)
	**≥ 1y**	**<200**	**2005**	1.9 (0.8,4.7)	1.9 (0.8,4.8)	2.2 (1.2,4.2)	2.3 (1.2,4.3)	1.7 (0.9,3.0)	2.0 (1.1,3.5)	4.6 (2.3,9.3)	4.0 (2.1,7.6)	2.0 (0.6,6.8)	3.1 (0.7,14.4)
			**2011**	1.2 (0.5,2.7)	1.1 (0.5,2.6)	1.4 (0.9,2.3)	1.3 (0.8,2.2)	1.1 (0.7,1.6)	1.2 (0.8,1.8)	3.0 (1.7,5.2)	2.3 (1.5,3.8)	0.9 (0.5,1.6)	1.4 (0.4,4.4)
			**2017**	0.9 (0.4,2.2)	1.2 (0.5,3.0)	1.1 (0.7,1.9)	1.4 (0.8,2.5)	0.8 (0.5,1.3)	1.2 (0.7,2.3)	2.3 (1.3,4.2)	2.4 (1.4,4.1)	0.2 (0.1,0.6)	0.3 (0.1,1.4)
		**≥1000**	**2005**	0.5 (0.2,1.3)	0.8 (0.3,2.0)	0.6 (0.3,1.2)	0.9 (0.5,1.7)	0.5 (0.2,0.8)	0.8 (0.4,1.6)	1.2 (0.6,2.6)	1.6 (0.8,2.9)	0.5 (0.1,2.2)	0.8 (0.1,4.2)
			**2011**	0.3 (0.1,0.7)	0.4 (0.2,1.1)	0.4 (0.2,0.6)	0.5 (0.3,0.9)	0.3 (0.2,0.5)	0.5 (0.3,0.9)	0.8 (0.4,1.4)	0.9 (0.6,1.5)	0.2 (0.1,0.5)	0.3 (0.1,1.2)
			**2017**	0.3 (0.1,0.6)	0.5 (0.2,1.4)	0.3 (0.2,0.5)	0.5 (0.3,1.1)	0.2 (0.1,0.4)	0.5 (0.2,1.1)	0.6 (0.3,1.1)	1.0 (0.5,1.8)	0.1 (0.0,0.2)	0.1 (0.0,0.4)
**10-14**	**<6m**	**<200**	**2005**	22.3 (10.4,47.7)	17.3 (8.2,36.7)	11.0 (6.9,17.7)	10.7 (6.6,17.3)	14.3 (9.3,21.9)	12.6 (8.4,18.8)	41.3 (23.4,72.8)	30.2 (18.9,48.1)	14.2 (7.1,28.5)	7.1 (2.1,23.5)
			**2011**	12.0 (5.7,25.5)	10.4 (4.9,22.1)	5.9 (3.8,9.4)	6.4 (3.9,10.4)	7.7 (5.1,11.6)	7.5 (5.0,11.4)	22.3 (12.8,38.8)	18.1 (11.3,28.9)	10.9 (6.0,19.7)	5.4 (1.7,17.6)
			**2017**	5.2 (2.3,12.0)	6.3 (2.8,14.2)	2.6 (1.4,4.6)	3.9 (2.2,7.0)	3.3 (2.0,5.7)	4.6 (2.7,7.8)	9.7 (5.0,18.6)	11.0 (6.3,19.2)	4.3 (1.3,13.8)	2.1 (0.4,10.3)
		**≥1000**	**2005**	3.6 (1.7,8.0)	5.1 (2.3,11.2)	1.8 (1.1,3.0)	3.1 (1.8,5.5)	2.3 (1.4,3.8)	3.7 (2.2,6.2)	6.7 (3.6,12.4)	8.9 (5.2,15.2)	0.4 (0.1,1.8)	0.2 (0.0,1.1)
			**2011**	2.0 (0.9,4.2)	3.0 (1.4,6.7)	1.0 (0.6,1.6)	1.9 (1.1,3.3)	1.3 (0.8,2.0)	2.2 (1.3,3.7)	3.6 (2.0,6.5)	5.3 (3.1,9.1)	0.3 (0.1,1.2)	0.2 (0.0,0.8)
			**2017**	0.8 (0.4,2.0)	1.9 (0.8,4.4)	0.4 (0.2,0.8)	1.1 (0.6,2.3)	0.5 (0.3,1.0)	1.3 (0.7,2.6)	1.6 (0.8,3.1)	3.2 (1.7,6.2)	0.1 (0.0,0.7)	0.1 (0.0,0.4)
	**≥ 1y**	**<200**	**2005**	2.0 (0.8,4.9)	1.9 (0.8,4.8)	2.4 (1.3,4.4)	2.3 (1.2,4.3)	1.8 (1.0,3.2)	2.0 (1.2,3.5)	4.9 (2.4,9.8)	4.0 (2.1,7.7)	1.8 (0.5,6.3)	2.8 (0.6,13.3)
			**2011**	1.3 (0.6,2.8)	1.2 (0.5,2.6)	1.5 (0.9,2.4)	1.4 (0.8,2.2)	1.1 (0.7,1.7)	1.2 (0.8,1.8)	3.1 (1.8,5.4)	2.4 (1.5,3.8)	0.8 (0.4,1.5)	1.3 (0.4,3.9)
			**2017**	1.0 (0.4,2.3)	1.2 (0.5,3.0)	1.2 (0.7,1.9)	1.4 (0.8,2.5)	0.9 (0.6,1.4)	1.2 (0.7,2.2)	2.4 (1.3,4.4)	2.5 (1.5,4.1)	0.2 (0.1,0.5)	0.3 (0.1,1.2)
		**≥1000**	**2005**	0.5 (0.2,1.3)	0.8 (0.3,2.0)	0.6 (0.3,1.2)	0.9 (0.5,1.8)	0.5 (0.3,0.9)	0.8 (0.4,1.6)	1.3 (0.6,2.7)	1.6 (0.8,3.0)	0.5 (0.1,2.1)	0.7 (0.1,4.0)
			**2011**	0.3 (0.1,0.8)	0.5 (0.2,1.1)	0.4 (0.2,0.7)	0.5 (0.3,0.9)	0.3 (0.2,0.5)	0.5 (0.3,0.9)	0.8 (0.5,1.5)	0.9 (0.6,1.5)	0.2 (0.1,0.5)	0.3 (0.1,1.1)
			**2017**	0.3 (0.1,0.6)	0.5 (0.2,1.4)	0.3 (0.2,0.5)	0.6 (0.3,1.1)	0.2 (0.1,0.4)	0.5 (0.2,1.1)	0.6 (0.4,1.2)	1.0 (0.5,1.8)	0.0 (0.0,0.1)	0.1 (0.0,0.3)

m, months; y, years.

Web Appendix H: Fitted mortality rate ratios and model parameters, and implied temporal trends, in the adjusted analysis of African regions

The fitted models for the adjusted analysis are presented below in Tables H1 (CHIV younger than 5 years) and H2 (CHIV at least 5 years of age).

The primary adjusted results discussed in the main article (left columns) allow for a temporal trend in the mortality rates among those LTFU, which is assumed to be equal to that estimated in the unadjusted routine analysis (see Web Appendix E for how a time trend is included in the simulation of outcomes). Results without this time modification, i.e., assuming no trend in mortality rates in those LTFU and using the simulation model as directly obtained from fitting to the tracing study data, are also shown (right columns).

The temporal trends implied by the model coefficients for the calendar time splines (see Web Appendix E for the form of the splines) are summarized in Table H3.

Table H1: Mortality rate ratios, time trends, and inter-programme heterogeneity among CHIV on ART younger than 5 years, based on the multivariable analysis of the adjusted routine data for African regions

	With time modification		No time modification	
	Mortality rate ratio		Mortality rate ratio	
	Estimate (95% CI)	p-value	Estimate (95% CI)	p-value
**Sex**				
Male	Ref		Ref	
Female	0.82 (0.63,1.07)	0.136	0.80 (0.62,1.04)	0.101
**ART duration**				
<6 months	Ref		Ref	
≥6 months and <1 year	0.36 (0.29,0.46)	<0.001	0.39 (0.29,0.51)	<0.001
≥1 year	0.11 (0.05,0.28)	<0.001	0.13 (0.05,0.30)	<0.001
**Current age (whole years)**				
<1	4.65 (2.78,7.77)	<0.001	4.96 (3.18,7.76)	<0.001
1-2	2.12 (1.34,3.37)	0.001	2.14 (1.67,2.73)	<0.001
3-4	Ref		Ref	
**CD4 % at ART initiation (%) - for ART duration < 1 year**				
<5	Ref		Ref	
5-10	0.65 (0.59,0.71)	<0.001	0.64 (0.58,0.70)	<0.001
11-15	0.53 (0.47,0.61)		0.51 (0.45,0.59)	
16-20	0.47 (0.40,0.55)		0.45 (0.38,0.53)	
21-25	0.42 (0.35,0.51)		0.40 (0.33,0.49)	
25-30	0.39 (0.32,0.47)		0.37 (0.30,0.46)	
>30	0.37 (0.30,0.45)		0.35 (0.28,0.44)	
**CD4 % at ART initiation (%) - for ART duration ≥ 1 year**				
<5	Ref		Ref	
5-10	0.78 (0.60,1.00)	0.048	0.76 (0.59,0.96)	0.023
11-15	0.69 (0.48,1.00)		0.66 (0.47,0.94)	
16-20	0.64 (0.41,1.00)		0.61 (0.40,0.93)	
21-25	0.60 (0.36,1.00)		0.57 (0.35,0.93)	
25-30	0.57 (0.33,1.00)		0.54 (0.32,0.92)	
>30	0.55 (0.31,1.00)		0.52 (0.30,0.91)	
**Region - for ART duration < 1 year**				
Central Africa	0.55 (0.28,1.07)	0.078	0.58 (0.27,1.22)	0.151
East Africa	Ref		Ref	
Southern Africa	0.62 (0.40,0.98)	0.041	0.63 (0.38,1.07)	0.087
West Africa	1.04 (0.73,1.49)	0.813	1.07 (0.68,1.66)	0.779
**Region - for ART duration ≥ 1 year**				
Central Africa	0.56 (0.18,1.76)	0.323	0.58 (0.18,1.90)	0.367
East Africa	Ref		Ref	
Southern Africa	0.38 (0.22,0.68)	0.001	0.41 (0.23,0.75)	0.004
West Africa	0.80 (0.47,1.35)	0.397	0.81 (0.46,1.43)	0.469
	**Parameter estimate**		**Parameter estimate**	
	**Estimate (95% CI)**	**P-value**	**Estimate (95% CI)**	**P-value**
**Calendar year - for ART duration < 1 year**				
Spline basis function 1	-0.83 (-1.31,-0.35)	<0.001	-0.98 (-1.53,-0.42)	<0.001
Spline basis function 2	-1.08 (-1.76,-0.40)	0.002	-0.62 (-1.26,0.01)	0.054
**Calendar year - for ART duration ≥ 1 year**				
Spline basis function 1	-0.01 (-1.65,1.64)	0.992	0.07 (-1.57,1.70)	0.936
Spline basis function 2	-0.09 (-1.20,1.03)	0.880	0.27 (-0.84,1.38)	0.636
**Random effect variance**†				
All regions	0.00		0.00	
Additional for Southern Africa	0.33		0.32	

† Calculation of formal CIs and p-values hindered by estimates of 0 random effect variances for some simulations.

Table H2: Mortality rate ratios, time trends, and inter-programme heterogeneity among CHIV on ART at least 5 years old, based on the multivariable analysis of the adjusted routine data for African regions

	With time modification		No time modification	
	Mortality rate ratio		Mortality rate ratio	
	Estimate (95% CI)	p-value	Estimate (95% CI)	p-value
**Sex**				
Male	Ref		Ref	
Female	0.81 (0.61,1.09)	0.173	0.80 (0.60,1.06)	0.123
**ART duration**				
<6 months	Ref		Ref	
≥6 months and <1 year	0.40 (0.28,0.56)	<0.001	0.37 (0.29,0.47)	<0.001
≥1 year	0.12 (0.03,0.40)	<0.001	0.08 (0.03,0.22)	<0.001
**Current age (whole years)**				
5-9	Ref		Ref	
10-14	1.01 (0.91,1.13)	0.813	1.03 (0.92,1.14)	0.649
**CD4 count at ART initiation (cells/mm^3) - for ART duration < 1 year**				
<200	Ref		Ref	
200-349	0.56 (0.50,0.64)	<0.001	0.56 (0.50,0.64)	<0.001
350-499	0.44 (0.37,0.53)		0.44 (0.37,0.53)	
500-749	0.36 (0.28,0.45)		0.36 (0.28,0.45)	
750-999	0.29 (0.22,0.39)		0.29 (0.22,0.39)	
≥1000	0.25 (0.19,0.35)		0.25 (0.19,0.35)	
**CD4 count at ART initiation (cells/mm^3) - for ART duration ≥ 1 year**				
<200	Ref		Ref	
200-349	0.65 (0.55,0.76)	<0.001	0.65 (0.55,0.76)	<0.001
350-499	0.54 (0.43,0.68)		0.54 (0.43,0.68)	
500-749	0.46 (0.34,0.61)		0.46 (0.34,0.61)	
750-999	0.39 (0.28,0.56)		0.39 (0.28,0.56)	
≥1000	0.35 (0.24,0.52)		0.35 (0.24,0.52)	
**Region - for ART duration < 1 year**				
Central Africa	1.63 (0.76,3.49)	0.214	1.70 (0.79,3.65)	0.178
East Africa	Ref		Ref	
Southern Africa	1.14 (0.67,1.93)	0.624	1.14 (0.68,1.93)	0.615
West Africa	2.74 (1.52,4.92)	<0.001	2.81 (1.56,5.08)	<0.001
**Region - for ART duration ≥ 1 year**				
Central Africa	0.85 (0.37,1.95)	0.696	0.87 (0.37,2.04)	0.746
East Africa	Ref		Ref	
Southern Africa	0.85 (0.48,1.51)	0.091	0.91 (0.52,1.58)	0.083
West Africa	1.69 (0.92,3.10)	0.695	1.68 (0.93,3.04)	0.769
	**Parameter estimate**		**Parameter estimate**	
	**Estimate (95% CI)**	**P-value**	**Estimate (95% CI)**	**P-value**
**Calendar year - for ART duration < 1 year**				
Spline basis function 1	-1.12 (-1.76,-0.48)	<0.001	-1.07 (-1.71,-0.44)	<0.001
Spline basis function 2	-1.08 (-1.83,-0.32)	0.005	-0.49 (-1.47,0.49)	0.324
**Calendar year - for ART duration ≥ 1 year**				
Spline basis function 1	-1.38 (-2.70,-0.05)	0.041	-1.11 (-2.38,0.16)	0.087
Spline basis function 2	0.08 (-0.71,0.87)	0.843	0.37 (-0.40,1.14)	0.345
**Random effect variance**[†]				
All regions	0.20		0.20	
Additional for Southern/West Africa	0.05		0.04	

[†] Calculation of formal CIs and p-values hindered by estimates of 0 random effect variances for some simulations.

Table H3: Mortality rate ratios describing temporal trends among CHIV on ART after LTFU (reference year: 2005), controlling for sex, age, ART duration, CD4 at ART start, and region, based on the multivariable analysis of the adjusted routine data for African regions

Age group	Year	With time modification				No time modification			
		ART duration < 1 year		ART duration ≥ 1 year		ART duration < 1 year		ART duration ≥ 1 year	
		Estimate (95% CI)	p-value	Estimate (95% CI)	p-value	Estimate (95% CI)	p-value	Estimate (95% CI)	p-value
**Age < 5 years**	**2010**	0.73 (0.60,0.87)	0.001	1.00 (0.53,1.88)	0.992	0.69 (0.56,0.85)	0.001	1.03 (0.55,1.92)	0.936
	**2017**	0.41 (0.28,0.59)	0.001	0.95 (0.47,1.95)	0.892	0.49 (0.34,0.71)	0.001	1.19 (0.59,2.40)	0.637
**Age ≥ 5 years**	**2010**	0.65 (0.51,0.83)	0.001	0.59 (0.35,0.98)	0.041	0.66 (0.52,0.84)	0.001	0.65 (0.40,1.06)	0.087
	**2017**	0.36 (0.25,0.52)	0.001	0.61 (0.35,1.07)	0.085	0.51 (0.31,0.84)	0.009	0.80 (0.47,1.36)	0.403

Web Appendix I: Sensitivity Analyses

The results of the sensitivity analyses are presented in two parts below. The first section focuses on the impact of analysis or data decisions related to how to 'adjust' mortality estimates for unreported deaths. The second section focuses on the handling of missing CD4 data.

Adjustment of mortality rates to account for unreported deaths

The following sensitivity analyses were performed:

- Not including a time trend in the simulation of outcomes
- Simulating outcomes in those LTFU for 90 days following LTFU, instead of for 6 months
- Simulating outcomes in those LTFU for 1 year following LTFU, instead of for 6 months
- Moving all center database closures to be 1 year earlier – this ensured that we were not over-adjusting from identifying more CHIV as LTFU than they were, which would be the case if closure dates were mistakenly too late (that is, we mistakenly believed that records transferred to IeDEA were more complete for the latest time periods than they were). Essentially, compared to the primary analysis, all data points within the last year of reporting are discarded, per center contributing data to each treatment programme.

Figures I1 to I8 summarize results from these sensitivity analyses. Given the large number of covariate patterns, estimated mortalities are shown for selected covariate values. In each figure, mortality rates are shown by CD4 at ART start, for each of the four African regions (rows), for 2005 and 2017 (columns), and for high- and low-mortality groups (columns) as defined by ART duration, current age and sex, based on results from the primary adjusted analysis.

For CHIV younger than 5 years (Figures I1 to I4), the high-mortality group consists of male CHIV with ART durations less 6 months and aged less than a year. The low-mortality group consist of females with ART durations of at least 1 year and aged 3-4 years.

For CHIV at least 5 years old (Figures I5 to I8), the high-mortality group consists of male CHIV with ART durations less 6 months and aged 10-14 years. The low-mortality group consist of females with ART durations of at least 1 year and aged 5-9 years.

In each figure, three sets of mortality estimates are shown: (i) the primary unadjusted estimates (black dashed line); (ii) the primary adjusted estimates (red solid line); and (iii) the secondary adjusted estimates from the sensitivity analysis (blue solid line). To reduce clutter, 95% CIs are shown only for the sensitivity analysis results.

For each distinct sensitivity analysis, the ratio change in the mortality rate, from the primary adjusted analysis to the sensitivity analysis, is summarized in Table I1. Considering all combinations of covariates values and calendar years (2004-2017), and weighting each combination equally, the minimum, average, and maximum change are indicated.

Results from the sensitivity analyses suggest that estimated mortality rates are generally robust relative to their uncertainties. There was little impact of moving database closure dates earlier; when removing the time trend in the simulation model, adjusted mortality estimates became slightly higher in recent years and lower in earlier years; and the impact of adjusting was dampened and strengthened by simulating for 90 days and 1 year instead respectively.

Table I1. The multiplicative change in mortality rates (as a %), from the primary adjusted analysis to the sensitivity analysis, when considering all combinations of covariate values and years

	Age < 5 years			Age ≥ 5 years		
	Ratio change in mortality rate (%)			Ratio change in mortality rate (%)		
	Minimum	Average	Maximum	Minimum	Average	Maximum
No time trend after LTFU	78	97	133	74	96	133
Simulate for 90 days after LTFU	74	91	115	71	90	109
Simulate for 1 year after LTFU	89	113	155	90	113	146
Move database closure dates earlier	83	99	118	84	102	127

Figure I1. Model-fitted mortality rates among CHIV on ART younger than 5 years, either unadjusted (black dashed line), adjusted (red solid line), or adjusted but without allowing for a time trend in the simulation of outcomes (blue solid line). Deaths per 100 person years (y-axis) are shown by CD4 % at ART start (x-axis), for each of the four African regions (rows), for 2005 and 2017 (columns), and for high- and low-mortality groups (columns) as defined by ART duration, age and sex (high: ART < 6 months, age <1 year, male; low: ART ≥ 1 year, age 3-4 years, female).

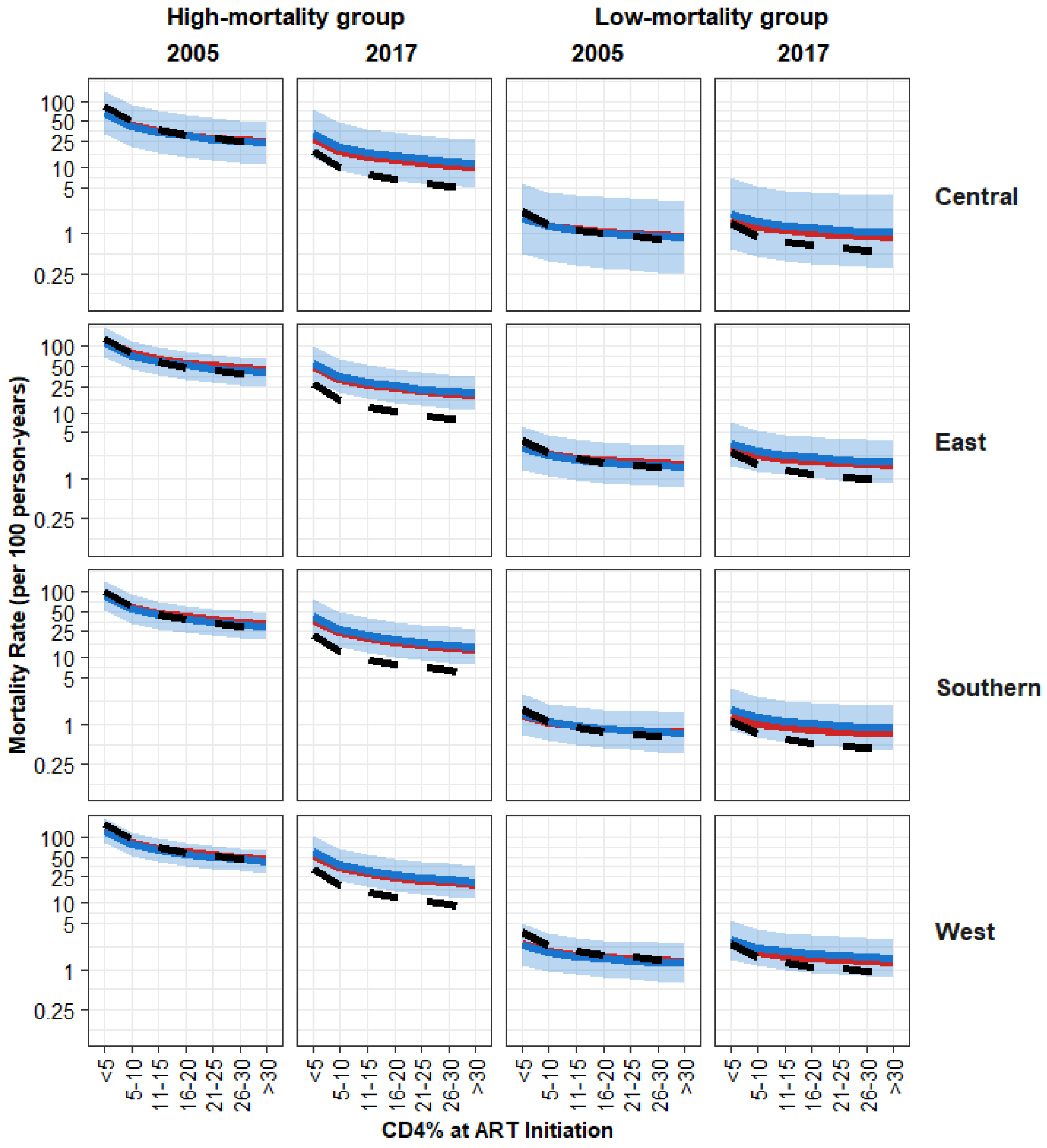

Figure I2. Model-fitted mortality rates among CHIV on ART younger than 5 years, either unadjusted (black dashed line), adjusted (red solid line), or adjusted but instead simulating outcomes for 90 days after LTFU (blue solid line). Deaths per 100 person-years (y-axis) are shown by CD4 % at ART start (x-axis), for each of the four African regions (rows), for 2005 and 2017 (columns), and for high- and low- mortality groups (columns) as defined by ART duration, age and sex (high: ART < 6 months, age <1 year, male; low: ART ≥ 1 year, age 3-4 years, female).

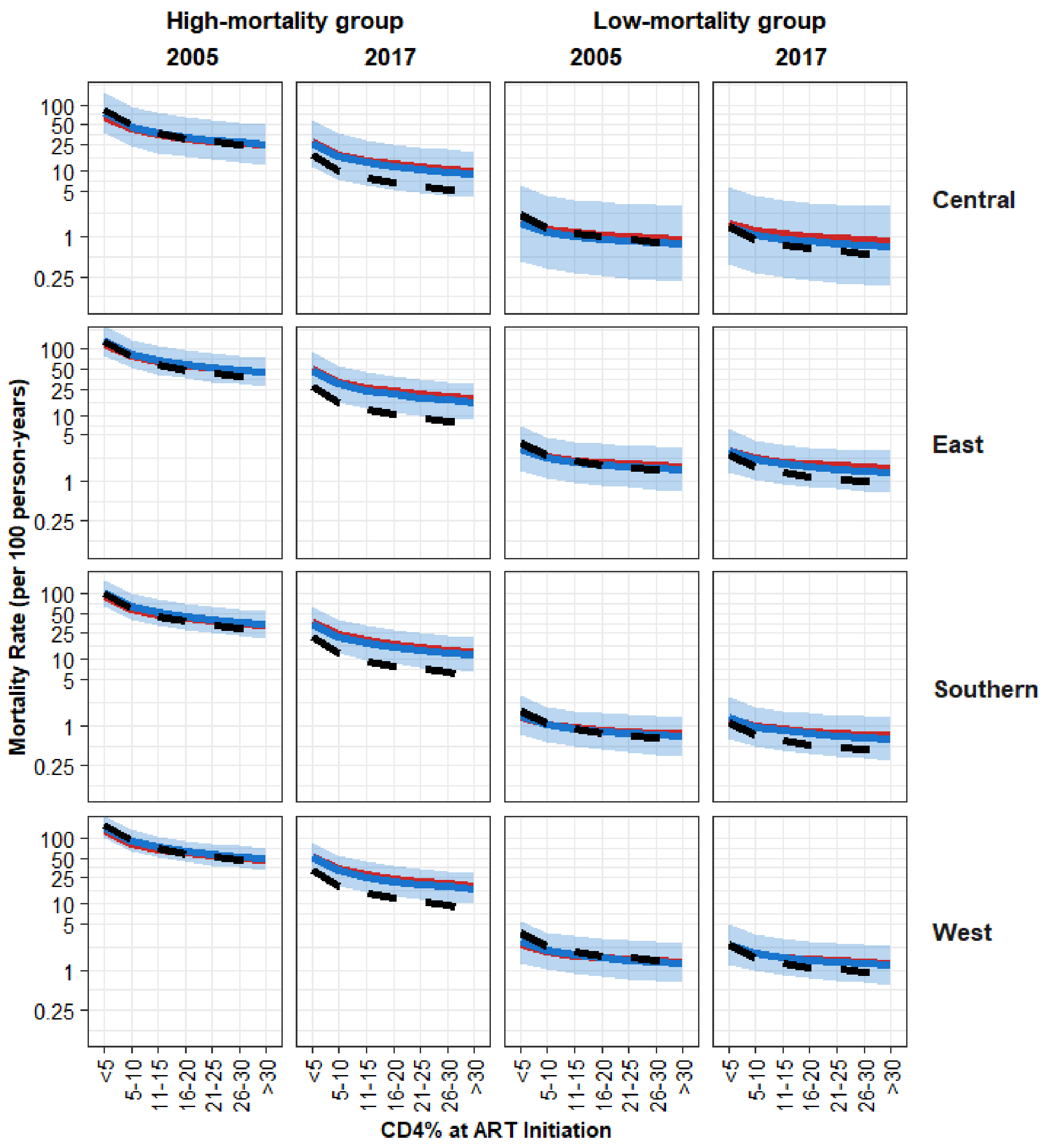

Figure I3. Model-fitted mortality rates among CHIV on ART younger than 5 years, either unadjusted (black dashed line), adjusted (red solid line), or adjusted but instead simulating outcomes for 1 year after LTFU (blue solid line). Deaths per 100 person-years (y-axis) are shown by CD4 % at ART start (x-axis), for each of the four African regions (rows), for 2005 and 2017 (columns), and for high- and low- mortality groups (columns) as defined by ART duration, age and sex (high: ART < 6 months, age <1 year, male; low: ART ≥ 1 year, age 3-4 years, female).

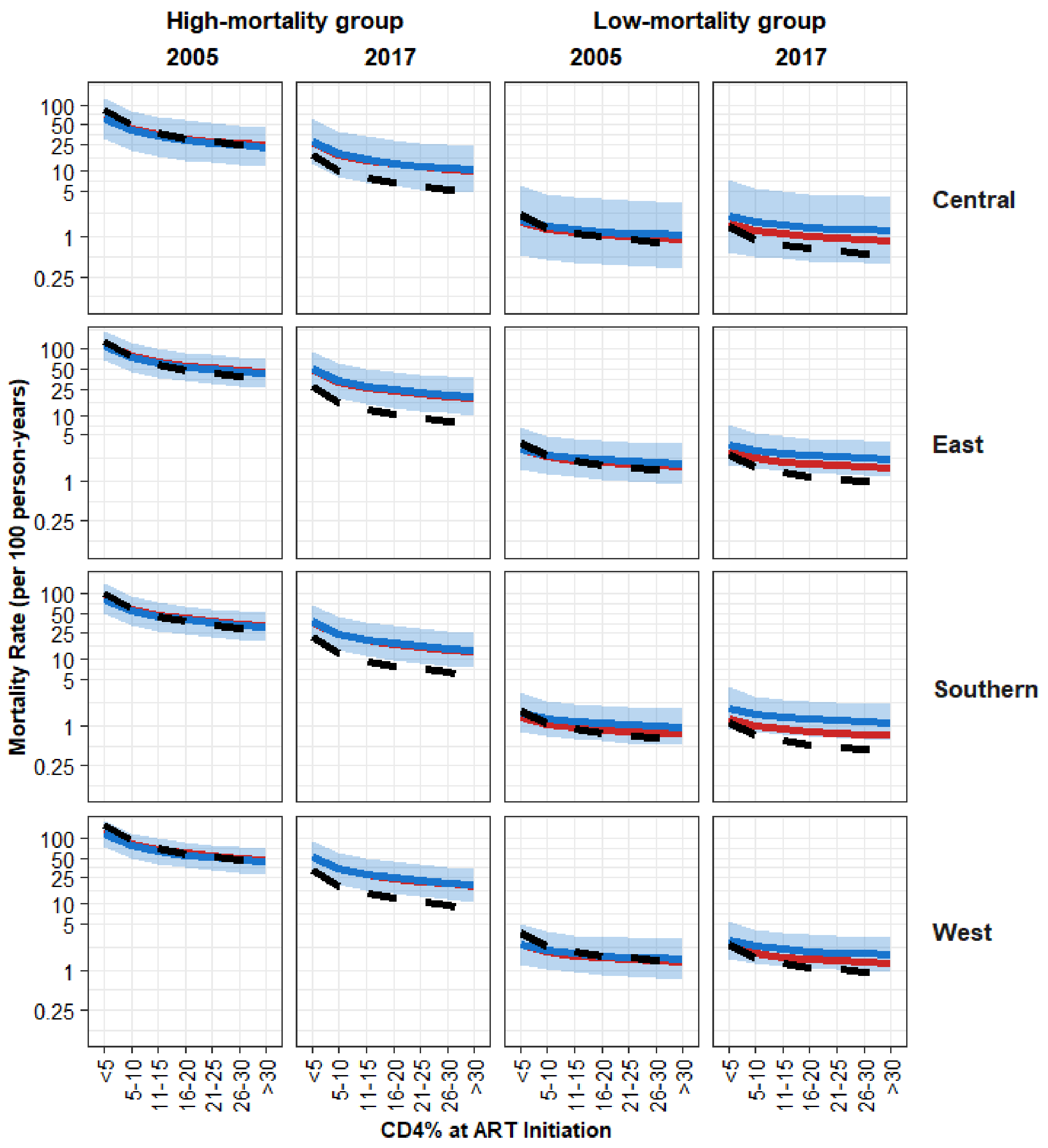

Figure I4. Model-fitted mortality rates among CHIV on ART younger than 5 years, either unadjusted (black dashed line), adjusted (red solid line), or adjusted but after moving all database closure dates forward by 1 year (blue solid line). Deaths per 100 person-years (y-axis) are shown by CD4 % at ART start (x-axis), for each of the four African regions (rows), for 2005 and 2017 (columns), and for high- and low- mortality groups (columns) as defined by ART duration, age and sex (high: ART < 6 months, age <1 year, male; low: ART ≥ 1 year, age 3-4 years, female).

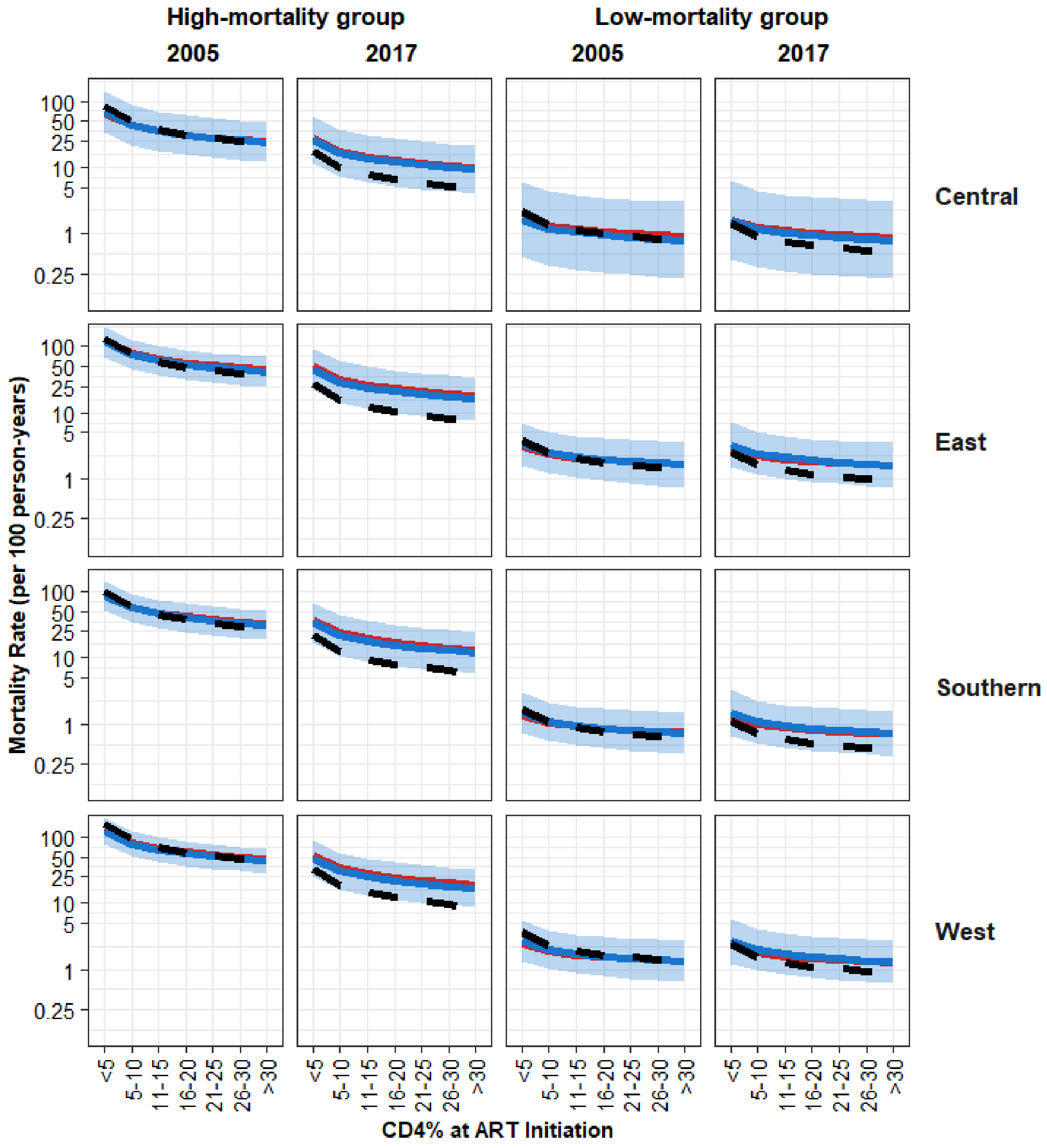

Figure I5. Model-fitted mortality rates among CHIV on ART at least 5 years old, either unadjusted (black dashed line), adjusted (red solid line), or adjusted but without allowing for a time trend in the simulation of outcomes (blue solid line). Deaths per 100 person-years (y-axis) are shown by CD4 count at ART start (x-axis), for each of the four African regions (rows), for 2005 and 2017 (columns), and for high- and low- mortality groups (columns) as defined by ART duration, age and sex (high: ART < 6 months, age 10-14 years, male; low: ART ≥ 1 year, age 5-9 years, female).

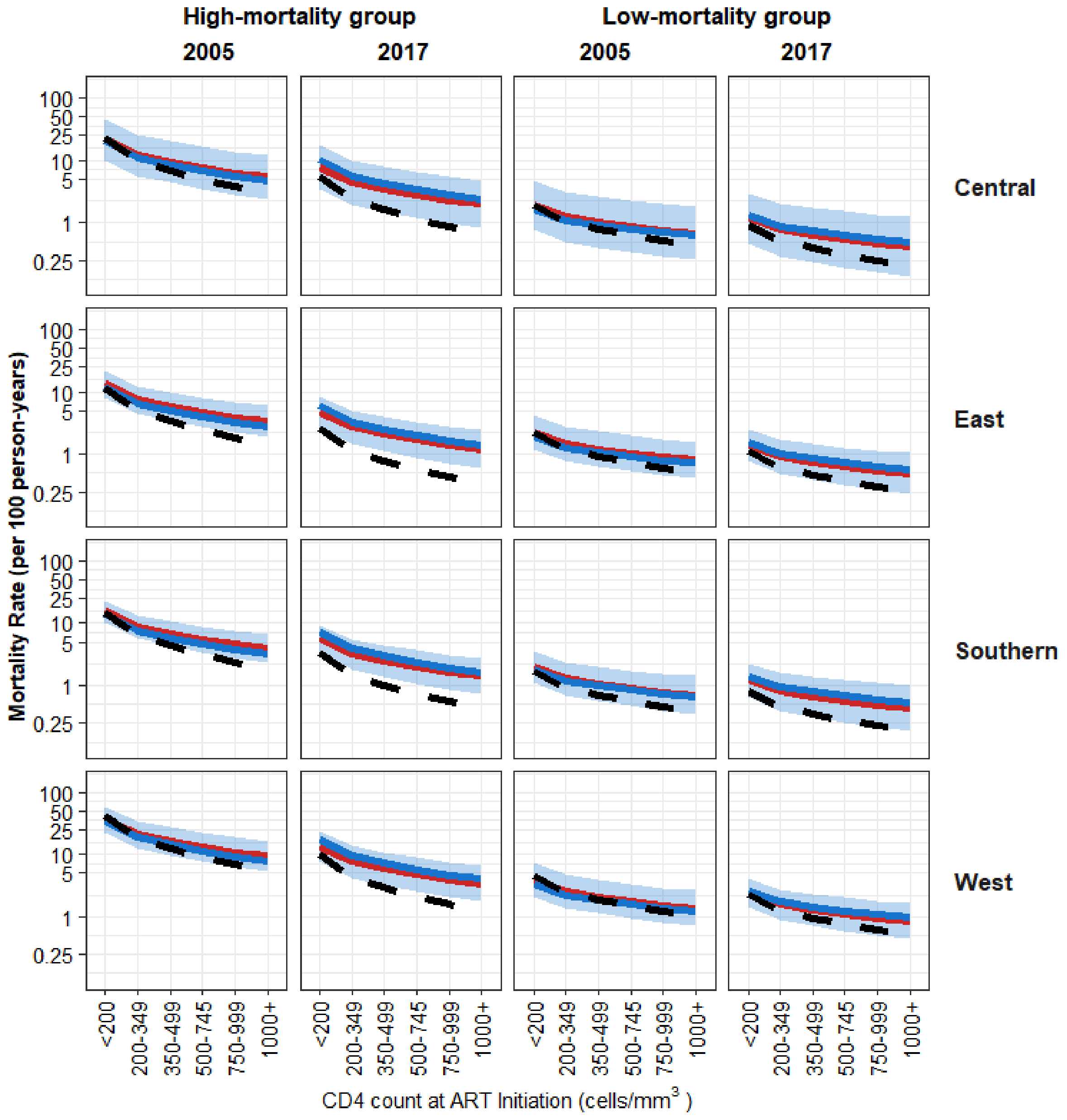

Figure I6. Model-fitted mortality rates among CHIV on ART at least 5 years old, either unadjusted (black dashed line), adjusted (red solid line), or adjusted but instead simulating outcomes for 90 days after LTFU (blue solid line). Deaths per 100 person-years (y-axis) are shown by CD4 count at ART start (x-axis), for each of the four African regions (rows), for 2005 and 2017 (columns), and for high- and low- mortality groups (columns) as defined by ART duration, age and sex (high: ART < 6 months, age 10-14 years, male; low: ART ≥ 1 year, age 5-9 years, female).

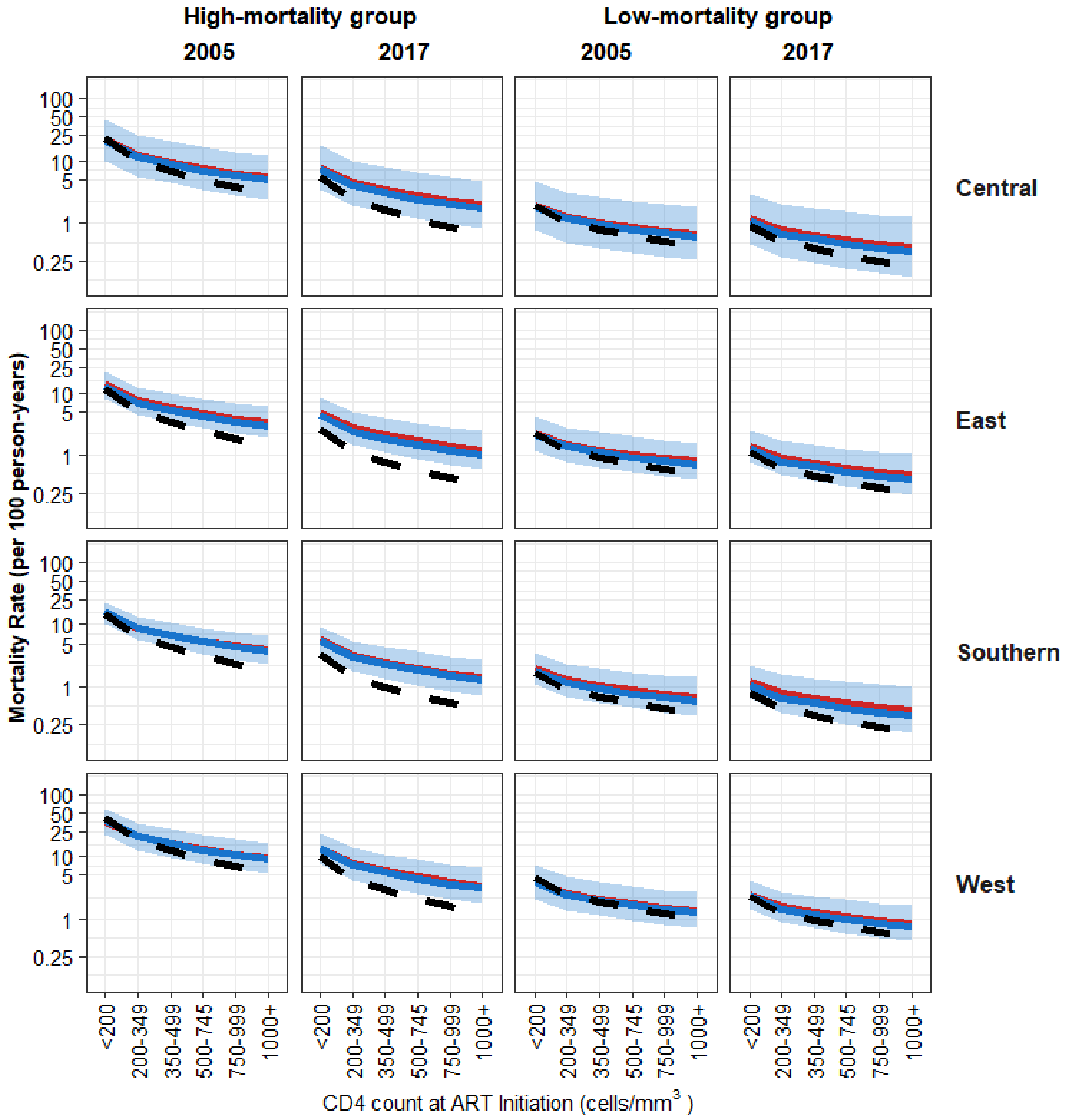

Figure I7. Model-fitted mortality rates among CHIV on ART at least 5 years old, either unadjusted (black dashed line), adjusted (red solid line), or adjusted but instead simulating outcomes for 1 year after LTFU (blue solid line). Deaths per 100 person-years (y-axis) are shown by CD4 count at ART start (x-axis), for each of the four African regions (rows), for 2005 and 2017 (columns), and for high- and low- mortality groups (columns) as defined by ART duration, age and sex (high: ART < 6 months, age 10-14 years, male; low: ART ≥ 1 year, age 5-9 years, female).

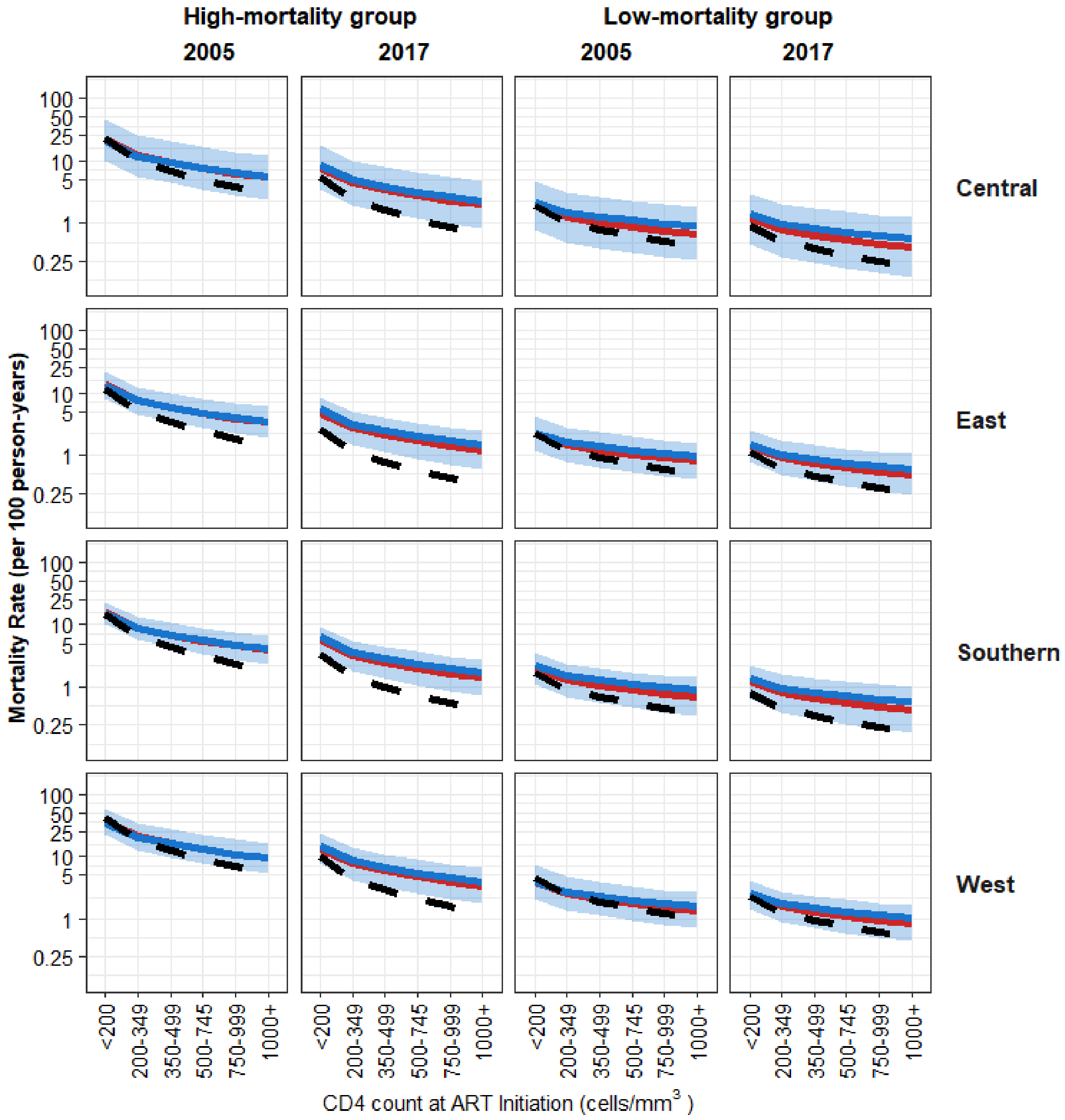

Figure I8. Model-fitted mortality rates among CHIV on ART at least 5 years old, either unadjusted (black dashed line), adjusted (red solid line), or adjusted but after moving all database closure dates forward by 1 year (blue solid line).. Deaths per 100 person-years (y-axis) are shown by CD4 count at ART start (x-axis), for each of the four African regions (rows), for 2005 and 2017 (columns), and for high- and low-mortality groups (columns) as defined by ART duration, age and sex (high: ART < 6 months, age 10-14 years, male; low: ART ≥ 1 year, age 5-9 years, female).

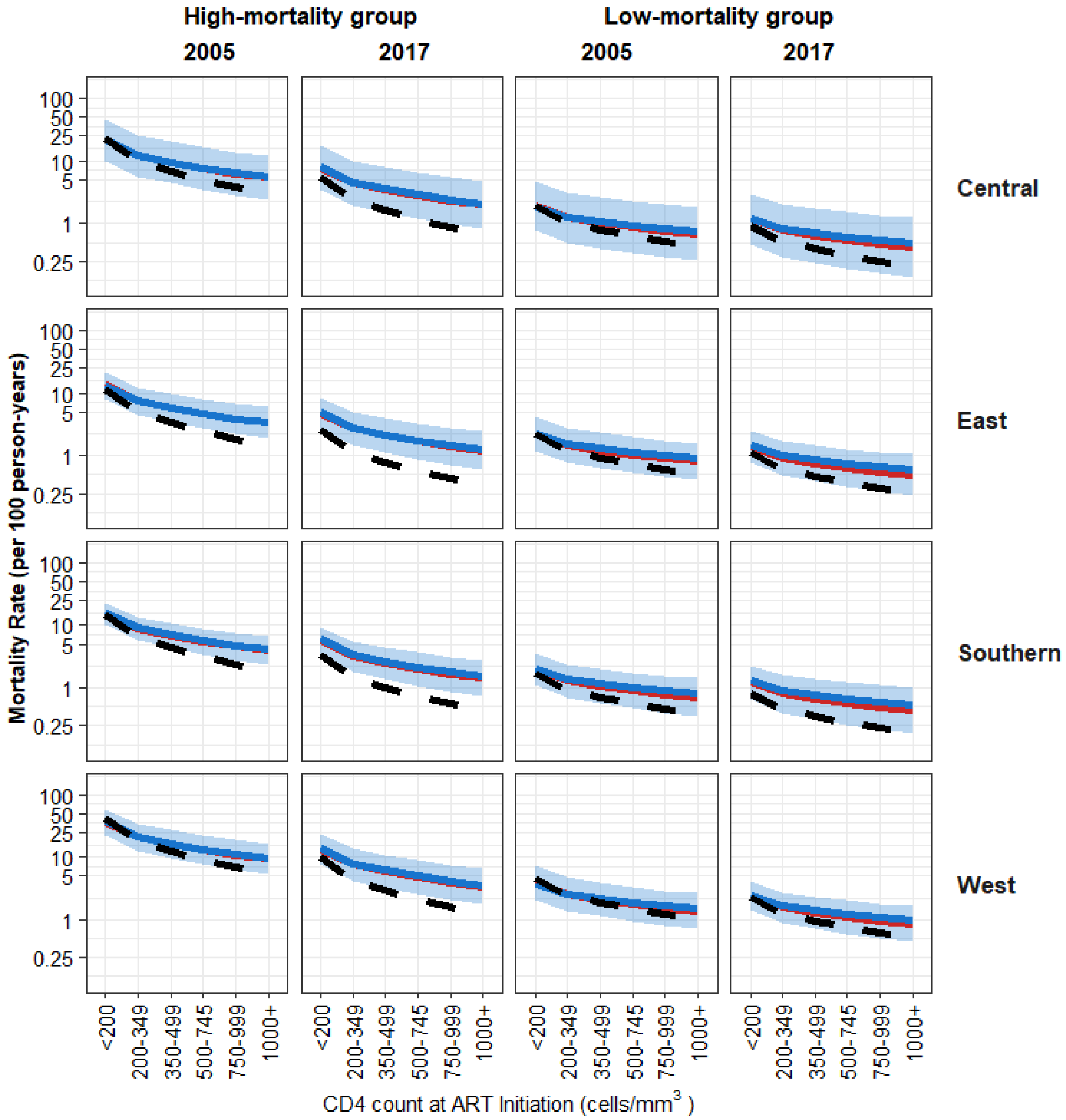

Handling of missing CD4 data

Almost no CD4 data are available in the tracing study dataset, and we therefore did not attempt to impute CD4 values for the tracing study analysis. The consequent limitations of our adjusted estimates remain (see Discussion of article).

In this sensitivity analysis, we chose to focus on the impact of imputing CD4 values for the routine data on the unadjusted mortality results.

Imputation of CD4 values

The MICE package in R was used to perform multiple imputation of CD4 values, by multivariate imputation by chained equations[4]. Predictive mean matching of CD4 values, built on a regression of the continuous CD4 values (on a log scale) on the imputation model predictors, was applied. Twenty imputed datasets were created, and results were combined using Rubin's rules[5]. Since no other variables used in the imputation model contained missing values, chains converged immediately.

Consistent with the analysis, separate imputation models were fitted for (i) African regions and (ii) the remaining regions. Separate models were also fitted to CHIV who started ART (i) before 5 years of age, specifically imputing CD4 percentage values, and (ii) when at least 5 years old, imputing CD4 count values. The extent of missing data in each of the four resulting data subsets is indicated in Table I2.

Imputation model predictors were sex, age at time of enrolment into programme, ART duration at time of enrolment, year of enrolment (quadratic polynomial), programme, time at risk (i.e., time from enrolment to death/censoring), whether time at risk ends with death or censoring, and whether the child is considered LTFU at censoring. A number of two-way interactions of the terms above were included, and time at risk was also split by whether the current age was less than 5 years or at least 5 years, and whether the current ART duration was less than 1 year versus at least 1 year (removing terms as needed to avoid collinearities).

Table I2: Percentage of CHIV with missing CD4 values

Regions	Age at ART start	Percentage of CHIV with missing CD4 values[†] (n[‡])
**African**	**< 5 years**	53 (40 681)
	**≥ 5 years**	40 (40 830)
**Other**	**< 5 years**	25 (2 505)
	**≥ 5 years**	10 (2 236)

[†] CD4 percent values for ART start age < 5 years, CD4 count values for ART start age ≥ 5 years. [‡] Sample sizes are larger than reported elsewhere since all CHIV, including those with missing CD4 values, are now considered.

[4] Reference: van Buuren S, Groothuis-Oudshoorn K. mice: Multivariate imputation by chained equations in R. Journal of Statistical Software 2011.

[5] Reference: Rubin DB. Multiple imputation for nonresponse in surveys. New York: John Wiley & Sons, Inc; 1987.

Comparison of fitted mortality rate ratios and estimated mortality rates

Tables I3 and I4 tabulate the model-fitted mortality rate ratios when excluding CHIV with missing CD4 values, and when imputing CD4 values as described above. Differences are typically small compared to uncertainty, however, the differences in mortality by region can sometimes be impacted more noticeably, as well as the time trends.

Table I5 summarises the changes in estimated mortalities, when considering the different combinations of covariates values and calendar years (2004-2017). The largest change in average mortality is a reduction of 12% (for regions outside of Africa, current age ≥ 5 years – driven mainly by a reduction in mortality rates for Latin America), and the changes can be large per individual covariate pattern.

Further studies are required to understand the CD4 missingness mechanisms, and thus how best to construct an imputation model of high predictive value.

Table I3: Mortality rate ratios among CHIV on ART younger than 5 years, based on the multivariable analysis of the unadjusted routine data, excluding CHIV with missing CD4 values and when imputing CD4 values

	Mortality rate ratio - estimate and 95% CI			
	Asia-Pacific and Latin America		African regions	
	Excluding CHIV with missing CD4	Imputing CD4 values	Excluding CHIV with missing CD4[‡]	Imputing CD4 values
**Sex**				
Male	Ref	Ref	Ref	Ref
Female	0.94 (0.66,1.32)	1.05 (0.79,1.41)	0.94 (0.84,1.05)	0.94 (0.87,1.02)
**ART duration**				
<6 months	Ref	Ref	Ref	Ref
≥6 months and <1 year	0.34 (0.21,0.54)	0.32 (0.21,0.49)	0.37 (0.31,0.43)	0.36 (0.32,0.40)
≥1 year	0.04 (0.01,0.29)	0.03 (0.01,0.21)	0.15 (0.06,0.36)	0.15 (0.07,0.30)
**Current age (whole years)**				
<1	4.79 (2.88,7.99)	6.06 (3.79,9.69)	5.63 (4.68,6.78)	4.67 (4.07,5.34)
1-2	1.75 (1.17,2.61)	2.19 (1.55,3.11)	2.24 (1.93,2.60)	2.43 (2.18,2.71)
3-4	Ref	Ref	Ref	Ref
**CD4 % at ART initiation (%) - for ART duration < 1 year**				
<5	Ref	Ref	Ref	Ref
5-10	0.30 (0.24,0.38)	0.31 (0.24,0.39)	0.57 (0.52,0.63)	0.58 (0.53,0.63)
11-15	0.17 (0.12,0.24)	0.18 (0.12,0.26)	0.44 (0.38,0.51)	0.45 (0.39,0.51)
16-20	0.12 (0.08,0.18)	0.12 (0.08,0.19)	0.37 (0.32,0.44)	0.38 (0.32,0.45)
21-25	0.09 (0.06,0.15)	0.09 (0.06,0.16)	0.33 (0.27,0.40)	0.33 (0.28,0.40)
25-30	0.07 (0.04,0.12)	0.08 (0.04,0.13)	0.30 (0.24,0.36)	0.30 (0.25,0.37)
>30	0.06 (0.04,0.11)	0.06 (0.03,0.11)	0.27 (0.22,0.34)	0.28 (0.22,0.35)
**CD4 % at ART initiation (%) - for ART duration ≥ 1 year**				
<5	Ref	Ref	Ref	Ref
5-10	0.50 (0.25,0.99)	0.45 (0.23,0.89)	0.65 (0.51,0.83)	0.63 (0.51,0.77)
11-15	0.36 (0.13,0.99)	0.31 (0.12,0.84)	0.53 (0.37,0.75)	0.51 (0.37,0.69)
16-20	0.29 (0.09,0.99)	0.25 (0.07,0.81)	0.47 (0.30,0.71)	0.44 (0.30,0.64)
21-25	0.25 (0.06,0.98)	0.20 (0.05,0.79)	0.42 (0.26,0.68)	0.39 (0.26,0.60)
25-30	0.22 (0.05,0.98)	0.18 (0.04,0.77)	0.39 (0.23,0.66)	0.36 (0.23,0.57)
>30	0.20 (0.04,0.98)	0.16 (0.03,0.76)	0.36 (0.21,0.64)	0.34 (0.21,0.55)
**Region - for ART duration < 1 year**				
Central Africa			0.89 (0.22,3.59)	1.00 (0.39,2.51)
East Africa			Ref	Ref
Southern Africa			0.74 (0.29,1.86)	0.84 (0.46,1.54)
West Africa			1.62 (0.60,4.39)	1.58 (0.78,3.20)
Asia-Pacific	Ref	Ref		
Latin America	0.99 (0.24,4.03)	0.73 (0.23,2.31)		
**Region - for ART duration ≥ 1 year**				
Central Africa			0.77 (0.14,4.29)	0.88 (0.27,2.84)
East Africa			Ref	Ref
Southern Africa			0.42 (0.16,1.10)	0.62 (0.33,1.17)
West Africa			1.29 (0.44,3.77)	1.05 (0.48,2.29)
Asia-Pacific	Ref	Ref		
Latin America	1.10 (0.15,8.26)	1.37 (0.31,5.98)		
**Time trend[†] - for ART duration < 1 year**				
2005	Ref	Ref	Ref	Ref
2017	0.31 (0.13,0.73)	0.35 (0.17,0.71)	0.21 (0.15,0.29)	0.28 (0.23,0.34)
**Time trend[†] - for ART duration ≥ 1 year**				
2005	Ref	Ref	Ref	Ref
2017	0.37 (0.03,5.27)	0.65 (0.08,5.30)	0.66 (0.34,1.27)	0.83 (0.52,1.33)

[†] The calendar time covariate is parameterized as before – only the change from 2005 to 2017 is reported in the table. [‡] Due to the computational requirements, for the African regions, the random effect structure was constrained to allow for a single variance value for all regions – there may therefore be small differences in mortality rate ratios from those reported elsewhere.

Table I4: Mortality rate ratios among CHIV on ART at least 5 years old, based on the multivariable analysis of the adjusted routine data, excluding CHIV with missing CD4 values and when imputing CD4 values

	Mortality rate ratio - estimate and 95% CI			
	Asia-Pacific and Latin America		African regions	
	Excluding CHIV with missing CD4	Imputing CD4 values	Excluding CHIV with missing CD4‡	Imputing CD4 values
**Sex**				
Male	Ref	Ref	Ref	Ref
Female	0.87 (0.64,1.19)	0.87 (0.65,1.17)	0.97 (0.87,1.08)	0.97 (0.88,1.06)
**ART duration**				
<6 months	Ref	Ref	Ref	Ref
≥6 months and <1 year	0.24 (0.14,0.41)	0.23 (0.14,0.39)	0.33 (0.27,0.39)	0.35 (0.30,0.41)
≥1 year	0.00 (0.00,0.05)	0.00 (0.00,0.03)	0.07 (0.03,0.18)	0.05 (0.02,0.12)
**Current age (whole years)**				
5-9	Ref	Ref	Ref	Ref
10-14	0.92 (0.65,1.30)	0.94 (0.67,1.31)	1.06 (0.94,1.18)	1.01 (0.91,1.11)
**CD4 count at ART initiation (cells/mm3) - for ART duration < 1 year**				
<200	Ref	Ref	Ref	Ref
200-349	0.19 (0.10,0.35)	0.18 (0.10,0.34)	0.43 (0.38,0.48)	0.41 (0.37,0.46)
350-499	0.09 (0.04,0.23)	0.09 (0.04,0.22)	0.30 (0.25,0.35)	0.28 (0.24,0.33)
500-749	0.05 (0.02,0.15)	0.05 (0.01,0.14)	0.22 (0.18,0.27)	0.20 (0.17,0.25)
750-999	0.03 (0.01,0.11)	0.03 (0.01,0.10)	0.16 (0.13,0.21)	0.15 (0.12,0.19)
≥1000	0.02 (0.00,0.08)	0.02 (0.00,0.08)	0.13 (0.10,0.17)	0.12 (0.09,0.16)
**CD4 count at ART initiation (cells/mm3) - for ART duration ≥ 1 year**				
<200	Ref	Ref	Ref	Ref
200-349	0.53 (0.36,0.78)	0.54 (0.38,0.76)	0.55 (0.49,0.61)	0.55 (0.49,0.61)
350-499	0.40 (0.23,0.70)	0.41 (0.25,0.67)	0.42 (0.36,0.50)	0.42 (0.36,0.49)
500-749	0.31 (0.16,0.63)	0.33 (0.18,0.61)	0.34 (0.27,0.41)	0.33 (0.27,0.41)
750-999	0.25 (0.11,0.58)	0.27 (0.13,0.55)	0.28 (0.22,0.35)	0.27 (0.22,0.34)
≥1000	0.22 (0.09,0.55)	0.23 (0.10,0.52)	0.24 (0.18,0.31)	0.23 (0.18,0.30)
**Region - for ART duration < 1 year**				
Central Africa			1.90 (0.67,5.32)	1.92 (0.77,4.79)
East Africa			Ref	Ref
Southern Africa			1.24 (0.66,2.34)	1.23 (0.66,2.30)
West Africa			3.61 (1.75,7.42)	3.92 (1.90,8.09)
Asia-Pacific	Ref	Ref		
Latin America	0.50 (0.15,1.60)	0.40 (0.12,1.36)		
**Region - for ART duration ≥ 1 year**				
Central Africa			0.79 (0.27,2.32)	1.04 (0.40,2.68)
East Africa			Ref	Ref
Southern Africa			0.72 (0.38,1.37)	0.90 (0.48,1.68)
West Africa			1.99 (0.96,4.13)	2.40 (1.16,4.98)
Asia-Pacific	Ref	Ref		
Latin America	1.57 (0.50,4.97)	1.12 (0.34,3.69)		
**Time trend† - for ART duration < 1 year**				
2005	Ref	Ref	Ref	Ref
2017	0.30 (0.10,0.91)	0.25 (0.08,0.76)	0.23 (0.16,0.35)	0.27 (0.20,0.36)
**Time trend† - for ART duration ≥ 1 year**				
2005	Ref	Ref	Ref	Ref
2017	0.10 (0.03,0.39)	0.15 (0.04,0.53)	0.49 (0.31,0.77)	0.55 (0.37,0.82)

† The calendar time covariate is parameterized as before – only the change from 2005 to 2017 is reported in the table. ‡ Due to the computational requirements, for the African regions, the random effect structure was constrained to allow for a single variance value for all regions – there may therefore be small differences in mortality rate ratios from those reported elsewhere.

Table I5: The multiplicative change in mortality rates (as a %), from the primary unadjusted analysis to the CD4 missingness sensitivity analysis, when considering all combinations of covariate values and years

Regions	Current age	Ratio change in mortality rate (%)		
		Minimum	Average	Maximum
**African**	**< 5 years**	64	103	169
	**≥ 5 years**	72	100	140
**Other**	**< 5 years**	59	104	223
	**≥ 5 years**	59	88	147
