# Supplementary material for: Global HIV mortality trends among children on antiretroviral treatment corrected for under‐reported deaths: an updated analysis of the International epidemiology Databases to Evaluate AIDS collaboration
Source: J Int AIDS Soc. 2021 Sep 21;24(Suppl 5):e25780. doi: 10.1002/jia2.25780 (PMC8454681; doi:10.1002/jia2.25780)
Supplement: Supplementary file 2 — Appendix S2. Lists of additional funding acknowledgements, site investigators and cohorts, as referenced in Acknowledgements of the article. [file JIA2-24-e25780-s001.pdf]

Acknowledgements

These acknowledgments relate to the following article:

Reshma Kassanjee, Leigh Johnson, Elizabeth Zaniewski, Marie Ballif, Benedikt Christ, Constantin Yiannoutsos, Patience Nyakato, Sophie Demonde, Andrew Edmonds, Tavitiya Sudjaritruk, Jorge Pinto, Rachel Vreeman, Désiré Lucien Dahourou, Christelle Twizere, Azar Kariminia, James Carlucci, Charles Kasozi, Mary-Ann Davies, on behalf of the International Epidemiology Databases to Evaluate AIDS (IeDEA) Collaboration. Global HIV mortality trends among children on antiretroviral treatment corrected for under-reported deaths: An updated analysis of the International epidemiology Databases to Evaluate AIDS collaboration. *JIAS*.

IeDEA Asia-Pacific

The TREAT Asia Pediatric HIV Observational Database is an initiative of TREAT Asia, a program of amfAR, The Foundation for AIDS Research, with support from the US National Institutes of Health's National Institute of Allergy and Infectious Diseases, the Eunice Kennedy Shriver National Institute of Child Health and Human Development, National Cancer Institute, National Institute of Mental Health, National Institute on Drug Abuse, the National Heart, Lung, and Blood Institute, the National Institute on Alcohol Abuse and Alcoholism, the National Institute of Diabetes and Digestive and Kidney Diseases, and the Fogarty International Center, as part of the International Epidemiology Databases to Evaluate AIDS (IeDEA; U01AI069907). The Kirby Institute is funded by the Australian Government Department of Health and Ageing, and is affiliated with the Faculty of Medicine, UNSW Australia. The content of this publication is solely the responsibility of the authors and does not necessarily represent the official views of any of the governments or institutions mentioned above.

Site investigators and cohorts

PS Ly, V Khol, National Centre for HIV/AIDS, Dermatology and STDs, Phnom Penh, Cambodia; J Tucker, New Hope for Cambodian Children, Phnom Penh, Cambodia; N Kumarasamy, E Chandrasekaran, Chennai Antiviral Research and Treatment Clinical Research Site (CART CRS), VHS-Infectious Diseases Medical Centre, VHS, Chennai, India; A Kinikar, V Mave, S Nimkar, I Marbaniang, BJ Medical College and Sassoon General Hospitals, Maharashtra, India; DK Wati, D Vedaswari, IB Ramajaya, Sanglah Hospital, Udayana University, Bali, Indonesia; N Kurniati, D Muktiarti, Cipto Mangunkusumo – Faculty of Medicine Universitas Indonesia, Jakarta, Indonesia; SM Fong, M Lim, F Daut, Hospital Likas, Kota Kinabalu, Malaysia; NK Nik Yusoff, P Mohamad, Hospital Raja Perempuan Zainab II, Kelantan, Malaysia; TJ Mohamed, MR Drawis, Department of Pediatrics, Women IeDEA global funding acknowledgements Version 16 December 2020 4 and Children Hospital Kuala Lumpur, Kuala Lumpur, Malaysia; R Nallusamy, KC Chan, Penang Hospital, Penang, Malaysia; T Sudjaritruk, V Sirisanthana, L Aurpibul, Department of Pediatrics, Faculty of Medicine, and Research Institute for Health Sciences, Chiang Mai University, Chiang Mai, Thailand; P Ounchanum, R Hansudewechakul, S Denjanta, A Kongphonoi, Chiangrai Prachanukroh Hospital, Chiang Rai, Thailand; P Lumbiganon, P Kosalaraksa, P Tharnprisan, T Udomphanit, Division of Infectious Diseases, Department of Pediatrics, Faculty of Medicine, Khon Kaen University, Khon Kaen, Thailand; G Jourdain, PHPT-IRD UMI 174 (Institut de recherche pour le développement and Chiang Mai University), Chiang Mai, Thailand; T Puthanakit, S Anugulruengkit, W Jantarabenjakul, R Nadsasarn, Department of Pediatrics and Center of Excellence for Pediatric Infectious Diseases and Vaccines, Faculty of Medicine, Chulalongkorn University, Bangkok, Thailand; K Chokephaibulkit, K Lapphra, W Phongsamart, S Sricharoenchai, Department of Pediatrics, Faculty of Medicine Siriraj Hospital, Mahidol University, Bangkok, Thailand; KH Truong, QT Du, CH Nguyen, Children's Hospital 1, Ho Chi Minh City, Vietnam; VC Do, TM Ha, VT An Children's Hospital 2, Ho Chi Minh City, Vietnam; LV Nguyen, DTK Khu, AN Pham, LT Nguyen, National Hospital of Pediatrics, Hanoi, Vietnam; ON Le, Worldwide Orphans Foundation, Ho

Chi Minh City, Vietnam; AH Sohn, JL Ross, T Suwanlerk, TREAT Asia/amfAR - The Foundation for AIDS Research, Bangkok, Thailand; MG Law, A Kariminia, The Kirby Institute, UNSW Sydney, NSW, Australia.

Caribbean, Central and South America network for HIV epidemiology (CCASAnet)

This work was supported by the NIH-funded Caribbean, Central and South America network for HIV epidemiology (CCASAnet), a member cohort of the International Epidemiologic Databases to Evaluate AIDS (leDEA) (U01AI069923). The content is solely the responsibility of the authors and does not necessarily represent the official views of the National Institutes of Health. This award is funded by the following institutes: the National Institute of Allergy and Infectious Diseases (NIAID), the Eunice Kennedy Shriver National Institute of Child Health and Human Development (NICHD), the National Cancer Institute (NCI), the National Institute of Mental Health (NIMH), the National Institute on Drug Abuse (NIDA), the National Heart, Lung, and Blood Institute (NHLBI), the National Institute on Alcohol Abuse and Alcoholism (NIAAA), the National Institute of Diabetes and Digestive and Kidney Diseases (NIDDK), the Fogarty International Center (FIC), and the National Library of Medicine (NLM).

Site investigators and cohorts

Fundación Huésped, Argentina: Pedro Cahn, Carina Cesar, Valeria Fink, Omar Sued, Emanuel Dell'Isola, Hector Perez, Jose Valiente, Cleyton Yamamoto.
Instituto Nacional de Infectologia-Fiocruz, Brazil: Beatriz Grinsztejn, Valdilea Veloso, Paula Luz, Raquel de Boni, Sandra Cardoso Wagner, Ruth Friedman, Ronaldo Moreira.
Universidade Federal de Minas Gerais, Brazil: Jorge Pinto, Flavia Ferreira, Marcelle Maia.
Universidade Federal de São Paulo, Brazil: Regina Célia de Menezes Succi, Daisy Maria Machado, Aida de Fátima Barbosa Gouvêa.
Fundación Arriarán, Chile: Marcelo Wolff, Claudia Cortes, Maria Fernanda Rodriguez, Gladys Allendes.
Les Centres GHESKIO, Haiti: Jean William Pape, Vanessa Rouzier, Adias Marcelin, Christian Perodin. **Hospital Escuela Universitario, Honduras:** Marco Tulio Luque.
Instituto Hondureño de Seguridad Social, Honduras: Denis Padgett.
Instituto Nacional de Ciencias Médicas y Nutrición Salvador Zubirán, Mexico: Juan Sierra Madero, Brenda Crabtree Ramirez, Paco Belaunzaran, Yanink Caro Vega.
Instituto de Medicina Tropical Alexander von Humboldt, Peru: Eduardo Gotuzzo, Fernando Mejia, Gabriela Carriquiry.
Vanderbilt University Medical Center, USA: Catherine C McGowan, Bryan E Shepherd, Timothy Sterling, Karu Jayathilake, Anna K Person, Peter F Rebeiro, Jessica Castilho, Stephany N Duda, Fernanda Maruri, Hilary Vansell, Cathy Jenkins, Ahra Kim, Sarah Lotspeich.

Central Africa IeDEA

Research reported in this publication was supported by the National Institutes of Health's National Institute of Allergy and Infectious Diseases (NIAID), the Eunice Kennedy Shriver National Institute of Child Health & Human Development (NICHD), the National Cancer Institute (NCI), the National Institute on Drug Abuse (NIDA), the National Heart, Lung, and Blood Institute (NHLBI), the National Institute on Alcohol Abuse and Alcoholism (NIAAA), the National Institute of Diabetes and Digestive and Kidney Diseases (NIDDK), the Fogarty International Center (FIC), the National Library of Medicine (NLM), and the Office of the Director (OD) under Award Number U01AI096299 (Central Africa-IeDEA). The content is solely the responsibility of the authors and does not necessarily represent the official views of the National Institutes of Health.

Site investigators and cohorts

Nimbona Pélagie, Association Nationale de Soutien aux Séropositifs et Malade du Sida (ANSS), Burundi; Patrick Gateretse, Jeanine Munezero, Valentin Nitereka, Théodore Niyongabo, Christelle Twizere, Centre National de Référence en Matière de VIH/SIDA, Burundi; Hélène Bukuru, Thierry Nahimana, Centre de Prise en Charge Ambulatoire et Multidisciplinaire des PVVIH/SIDA du Centre Hospitalo-Universitaire de Kamenge (CPAMPCHUK), Burundi; Elysée Baransaka, Patrice Barasukana, Eugene Kabanda, Martin Manirakiza, François Ndikumwenayo, CHUK/Burundi National University, Burundi; Jérémie Biziragusenyuka, Ange Marie Michelline Munezero, Centre de Prise en Charge Ambulatoire et Multidisciplinaire des PVVIH/SIDA de l'Hôpital Prince Régent Charles (CPAMP-HPRC), Burundi; Denis Nsame Nforniwe, Bamenda Hospital, Cameroon; Rogers Ajeh, Marc Lionel Ngamani, Clinical Research Education and Consultancy (CRENC), Cameroon; Anastase Dzudie, CRENC and Douala General Hospital, Cameroon; Akindeh Mbuh, CRENC and University of Yaoundé, Cameroon; Djenabou Amadou, Eric Walter Pefura Yone, Jamot Hospital, Cameroon; Ernestine Kendowo, Limbe Regional Hospital, Cameroon; Catherine Akele, Akili Clever, Faustin Kitetele, Patricia Lelo, Martine Tabala, Kalembelembe Pediatric Hospital, Democratic Republic of Congo; Cherubin Ekembe, Didine Kaba, Kinshasa School of Public Health, Democratic Republic of Congo; Merlin Diafouka, Martin Herbas Ekat, Dominique Mahambou Nsonde, CTA Brazzaville, Republic of Congo; Adolphe Mafoua, Massamba Ndala Christ, CTA Pointe-Noire, Republic of Congo; Jules Igirimbabazi, Nicole Ayinkamiye, Bethsaida Health Center, Rwanda; Providance Uwineza, Emmanuel Ndamijimana, Busanza Health Center, Rwanda; Emmanuel Habarurema, Marie Luise Nyiraneza, Gahanga Health Center, Rwanda; Marie Louise Nyiransabimana, Liliane Tuyisenge, Gikondo Health Center, Rwanda; Christian Shyaka, Catherine Kankindi, Kabuga Health Center, Rwanda; Bonheur Uwakijijwe, Marie Grace Ingabire, Kicukiro Health Center, Rwanda; Jules Ndumuhire, Marie Goretti Nyirabahutu, Masaka Health Center, Rwanda; Fred Muyango, Jean Christophe Bihibindi, Nyagasambu Health Center, Rwanda; Oliver Uwamahoro, Yvette Ndoli, Nyarugunga Health Center, Rwanda; Sabin Nsanzimana, Placidie Mugwaneza, Eric Remera, Esperance Umumararungu, Gallican Nshogoza Rwibasira, Dominique Savio Habimana, Rwanda Biomedical Center, Rwanda; Josephine Gasana, Faustin Kanyabwisha, Gallican Kubwimana, Benjamin Muhoza, Athanase Munyaneza, Gad Murenzi, Francoise Musabyimana, Francine Umwiza, Charles Ingabire, Patrick Tuyisenge, Alex M. Butera, Jules Kabahizi, Ephrem Rurangwa, Rwanda Military Hospital, Rwanda; Rosine Feza, Eugenie Mukashyaka, Shyorongi Health Center, Rwanda; Chantal Benekigeri, Jacqueline Musaninyange, WE-ACTx Health Center, Rwanda.

Coordinating and Data Centers

Adebola Adedimeji, Kathryn Anastos, Madeline Dilorenzo, Lynn Murchison, Jonathan Ross, Marcel Yotebieng, Albert Einstein College of Medicine, USA; Diane Addison, Ellen Brazier, Heidi Jones, Elizabeth Kelvin, Sarah Kulkarni, Denis Nash, Matthew Romo, Olga Tymejczyk, Institute for Implementation Science in Population Health, Graduate School of Public Health and Health Policy, City University of New York (CUNY), USA; Batya Elul, Columbia University, USA; Xiatao Cai, Allan Dong, Don Hoover, Hae-Young Kim, Chunshan Li, Qiuhu Shi, Data Solutions, USA; Kathryn Lancaster, The Ohio State University, USA; Mark Kuniholm, University at Albany, State University of New York, USA; Andrew Edmonds, Angela Parcesepe, Jess Edwards, University of North Carolina at Chapel Hill, USA; Olivia Keiser, University of Geneva; Stephany Duda; Vanderbilt University School of Medicine, USA; April Kimmel, Virginia Commonwealth University School of Medicine, USA.

East Africa IeDEA

Research reported in this publication was supported by the National Institute Of Allergy And Infectious Diseases (NIAID), Eunice Kennedy Shriver National Institute Of Child Health & Human Development (NICHD), National Institute On Drug Abuse (NIDA), National Cancer Institute (NCI), and the National Institute of Mental Health (NIMH), National Institute of Diabetes and Digestive and Kidney Diseases

(NIDDK) , Fogarty International Center (FIC), National Heart, Lung, and Blood Institute (NHLBI) , in accordance with the regulatory requirements of the National Institutes of Health under Award Number U01AI069911East Africa IeDEA Consortium. The content is solely the responsibility of the authors and does not necessarily represent the official views of the National Institutes of Health.

Site investigators, cohorts, data managers

Diero L, Ayaya S, Sang E, MOI University, AMPATH Plus, Eldoret, Kenya; Bukusi E, Edwin Mulwa, George Nyanaro, KEMRI (Kenya Medical Research Institute), Kisumu, Kenya; Charles Kasozi , Mathew Ssemakadde, Masaka Regional Referral Hospital, Masaka, Uganda; Mwebesa Bosco Bwana, Winnie Muyindike, Helen Byakwaga Michael Kanyesigye, Mbarara University of Science and Technology (MUST), Mbarara, Uganda; Barbara Castelnuovo, Aggrey Semeere,; John Michael Matovu, Infectious Diseases Institute (IDI), Mulago, Uganda; Fred Nalugoda, Francis X. Wasswa, Rakai Health Sciences Program, Kalisizo, Uganda; Paul Kazyoba, Mary Mayige, (NIMR), Dar es Salaam, Tanzania; Rita Elias Lyamuya, Francis Mayanga, Morogoro Regional Hospital, Morogoro, Tanzania; Kapella Ngonyani, Jerome Lwali, Tumbi Regional Hospital, Pwani, Tanzania; Mark Urassa, Charles Nyaga, Richard Machemba, National Institute for Medical Research (NIMR), Kisesa HDSS, Mwanza, Tanzania; Kara Wools-Kaloustian, Constantin Yiannoutsos, Beverly Musick, Indiana University School of Medicine, Indiana University, Indianapolis, IN, USA; Batya Elul, Columbia University, New York City, NY, USA; Rachel Vreeman (Mt. Sinai, New York, USA) Jennifer Syvertsen, (University of California Riverside, California, USA; Rami Kantor, Brown University/Miriam Hospital, Providence, RI, USA; Jeffrey Martin, Megan Wenger, Craig Cohen, Jayne Kulzer, University of California, San Francisco, CA, USA;

IeDEA Southern Africa

Research reported in this publication was supported by the U.S. National Institutes of Health’s National Institute of Allergy and Infectious Diseases, the Eunice Kennedy Shriver National Institute of Child Health and Human Development, the National Cancer Institute, the National Institute of Mental Health, the National Institute on Drug Abuse, the National Heart, Lung, and Blood Institute, the National Institute on Alcohol Abuse and Alcoholism, the National Institute of Diabetes and Digestive and Kidney Diseases and the Fogarty International Center under Award Number U01AI069924. The content is solely the responsibility of the authors and does not necessarily represent the official views of the National Institutes of Health.

Site investigators and cohorts

Gary Maartens, Aid for AIDS, South Africa; Carolyn Bolton, Centre for Infectious Disease Research in Zambia (CIDRZ), Zambia; Robin Wood, Gugulethu (Desmond Tutu HIV Centre), South Africa; Nosisa Sipambo, Harriet Shezi Children’s Clinic, South Africa; Frank Tanser, Hlabisa (Africa Health Research Institute), South Africa; Andrew Boulle, Khayelitsha ART Programme, South Africa; Geoffrey Fatti, Kheth’Impilo AIDS Free Living, South Africa; Sam Phiri, Lighthouse Trust, Malawi; Elvira Singh, National Cancer Registry (National Health Laboratory Service), South Africa; Cleophas Chimbetete, Newlands Clinic (Ruedi Luethy Foundation Zimbabwe), Zimbabwe; Karl Technau, Rahima Moosa Mother and Child Hospital, South Africa; Brian Eley, Red Cross War Memorial Children’s Hospital, South Africa; Josephine Muhairwe, SolidarMed Lesotho; Idivino Rafael, SolidarMed Mozambique; Cordelia Kunzekwenyika, SolidarMed Zimbabwe, Matthew P Fox, Themba Lethu Clinic, South Africa; Hans Prozesky, Tygerberg Hospital, South Africa.

Data centers

Nina Anderegg, Marie Ballif, Benedikt Christ, Cam Ha Dao Ostinelli, Matthias Egger, Lukas Fenner, Andreas Haas, Anthony Hauser, Stefanie Hossmann, Serra Lem, Catrina Mugglin, Radoslaw Panczak, Eliane Rohner, Julien Riou, Veronika Skrivankova, Lilian Smith, Katayoun Taghavi, Per von Groote, Gilles Wandeler, Elizabeth Zaniewski, Kathrin Zürcher, Institute of Social and Preventive Medicine, University of Bern, Switzerland; Kim Anderson, Andrew Boulle, Morna Cornell, Mary-Ann Davies,

Victoria Iyun, Leigh Johnson, Reshma Kassanjee, Kathleen Kehoe, Mmamapudi Kubjane, Nicola Maxwell, Patience Nyakato, Gem Patten, Mpho Tlali, Priscilla Tsondai, Renee de Waal, School of Public Health and Family Medicine, University of Cape Town, South Africa.

IeDEA West Africa

Research reported in this publication was supported by the US National Institutes of Health (NIAID, NICHD, NCI and NIMH) under Award Number U01AI069919 (PI: Dabis). The content is solely the responsibility of the authors and does not necessarily represent the official views of the National Institutes of Health.

Site investigators and cohorts

Adult cohorts: Marcel Djimon Zannou, CNHU, Cotonou, Benin; Armel Poda, CHU Souro Sanou, Bobo Dioulasso, Burkina Faso; Fred Stephen Sarfo & Komfo Anokeye Teaching Hospital, Kumasi, Ghana; Eugene Messou, ACONDA CePReF, Abidjan, Cote d'Ivoire; Henri Chenal, CIRBA, Abidjan, Cote d'Ivoire; Kla Albert Minga, CNTS, Abidjan, Cote d'Ivoire; Emmanuel Bissagnene, & Aristophane Tanon, CHU Treichville, Cote d'Ivoire; Moussa Seydi, CHU de Fann, Dakar, Senegal; Akessiwe Akouda Patassi, CHU Sylvanus Olympio, Lomé, Togo. Pediatric cohorts: Sikiratou Adouni Koumakpai-Adeothy,_CNHU, Cotonou, Benin; Lorna Awo Renner, Korle Bu Hospital, Accra, Ghana; Sylvie Marie N'Gbeche, ACONDA CePReF, Abidjan, Ivory Coast; Clarisse Amani Bosse, ACONDA_MTCT+, Abidjan, Ivory Coast; Kouadio Kouakou, CIRBA, Abidjan, Cote d'Ivoire; Madeleine Amorissani Folquet, CHU de Cocody, Abidjan, Cote d'Ivoire; François Tanoh Eboua, CHU de Yopougon, Abidjan, Cote d'Ivoire; Fatoumata Dicko Traore, Hopital Gabriel Toure, Bamako, Mali; Elom Takassi, CHU Sylvanus Olympio, Lomé,Togo

Coordinating and data centers

François Dabis, Elise Arrive, Eric Balestre, Renaud Becquet, Charlotte Bernard, Shino Chassagne Arikawa, Alexandra Doring, Antoine Jaquet, Karen Malateste, Elodie Rabourdin, Thierry Tiendrebeogo, ADERA, Isped & INSERM U1219, Bordeaux, France. Sophie Desmonde, Julie Jesson, Valeriane Leroy, Inserm 1027, Toulouse, France Didier Koumavi Ekouevi, Jean-Claude Azani, Patrick Coffie, Abdoulaye Cissé, Guy Gnepa, Apollinaire Horo, Christian Kouadio, Boris Tchounga, PACCI, CHU Treichville, Abidjan, Côte d'Ivoire

---
